# Genome-Wide Characterization of Jasmonates Signaling Components Reveals the Essential Role of ZmCOI1a-ZmJAZ15 Action Module in Regulating Maize Immunity to Gibberella Stalk Rot

**DOI:** 10.3390/ijms22020870

**Published:** 2021-01-16

**Authors:** Liang Ma, Yali Sun, Xinsen Ruan, Pei-Cheng Huang, Shi Wang, Shunfa Li, Yu Zhou, Fang Wang, Yu Cao, Qing Wang, Zhenhua Wang, Michael V. Kolomiets, Xiquan Gao

**Affiliations:** 1 State Key Laboratory for Crop Genetics and Germplasm Enhancement, Nanjing Agricultural University, Nanjing 210095, China; 2019201077@njau.edu.cn (L.M.); 2017201002@njau.edu.cn (Y.S.); 2017201073@njau.edu.cn (X.R.); shi.wang@nbmedicalsystem.com (S.W.); 2017101069@njau.edu.cn (S.L.); 2017101068@njau.edu.cn (F.W.); 2018101059@njau.edu.cn (Y.C.); qingwang@njau.edu.cn (Q.W.); 2 Jiangsu Collaborative Innovation Center for Modern Crop Production, Nanjing Agricultural University, Nanjing 210095, China; 3 College of Agriculture, Nanjing Agricultural University, Nanjing 210095, China; 4 Department of Plant Pathology and Microbiology, Texas A&M University, College Station, TX 77840-2132, USA; masonhuang0208@tamu.edu (P.-C.H.); kolomiets@tamu.edu (M.V.K.); 5 College of Agriculture, Northeast Agricultural University, Harbin 150030, China; zhouyu0924@126.com (Y.Z.); zhenhuawang_2006@163.com (Z.W.)

**Keywords:** coronatine, *Fusarium graminearum*, jasmonic acid, maize stalk rot, oxylipins

## Abstract

Gibberella stalk rot (GSR) by *Fusarium graminearum* causes significant losses of maize production worldwide. Jasmonates (JAs) have been broadly known in regulating defense against pathogens through the homeostasis of active JAs and COI-JAZ-MYC function module. However, the functions of different molecular species of JAs and COI-JAZ-MYC module in maize interactions with *Fusarium graminearum* and regulation of diverse metabolites remain unknown. In this study, we found that exogenous application of MeJA strongly enhanced resistance to GSR. RNA-seq analysis showed that MeJA activated multiple genes in JA pathways, which prompted us to perform a genome-wide screening of key JA signaling components in maize. Yeast Two-Hybrid, Split-Luciferase, and Pull-down assays revealed that the JA functional and structural mimic coronatine (COR) functions as an essential ligand to trigger the interaction between ZmCOIa and ZmJAZ15. By deploying CRISPR-cas9 knockout and *Mutator* insertional mutants, we demonstrated that *coi1a* mutant is more resistant, whereas *jaz15* mutant is more susceptible to GSR. Moreover, JA-deficient *opr7-5opr8-2* mutant displayed enhanced resistance to GSR compared to wild type. Together, these results provide strong evidence that *ZmJAZ15* plays a pivotal role, whereas *ZmCOIa* and endogenous JA itself might function as susceptibility factors, in maize immunity to GSR.

## 1. Introduction

In nature, plants are constantly encountering innumerable incursions by diverse environmental stresses, such as heat, drought, cold, insects, and pathogens. To retaliate against these foes, plants have evolved a plethora of strategies to sustain development and growth, while resisting these adverse abiotic and biotic stresses by activating multilayered defense responses. One of these strategies is to activate and reprogram the complex phytohormone signaling networks [1,2]. It has been well-documented that phytohormones play an important role in balancing growth and defense [3], among which two major hormones often associated with defense response are salicylic acid (SA) and jasmonates (JAs) [4,5].

JAs belong to a group of oxygenated fatty acid products, called oxylipins, derived from the enzymatic oxygenation of alfa-linolenic fatty acid by 13-Lipoxygenases (LOXs) and several other consecutive enzymes including allene oxide synthase (AOS), and allene oxide cyclase (AOC) to form 12-oxo-phytodienoic acid (12-OPDA), the precursor of JA. 12-OPDA is metabolized via 12-oxo-phytodienoic acid reductase (OPRs), and three cycles of beta-oxidation [6,7,8,9], to produce JA. Alfa-linolenic and linoleic acids also serve as the substrates to form 9-oxylipins, the diverse products of 9-LOXs-mediated oxidation of fatty acids at carbon position nine. JAs have been well documented for their vital roles in regulating plant growth and development, responses to mechanical trauma, and diverse abiotic and biotic stresses [3,7,9,10,11]. In particular, JAs display inhibitory effects on primary root growth [12,13,14]. On the other hand, JAs positively regulate stamen development [15], male and female fertility [16,17], anthocyanin biosynthesis [17,18], and defense against insects and necrotrophic pathogens [1,19]. Multiple roles of JA in maize are best illustrated in the study of the *opr7opr8* double mutant, which is deficient in JA biosynthesis, is male-sterile, lacks anthocyanin production, and is susceptible to soil-borne pathogen *Pythium* spp. [18] and *Fusarium verticillioides* [20], but resistant to anthracnose leaf blight and stalk rot, caused by a hemibiotrophic pathogen *Colletotrichum graminicola* [21]. Although underappreciated previously, 12-OPDA has been increasingly drawing attention as having functions distinct from those of JAs [22,23,24,25,26,27]. Particularly, a recent study revealed that 12-OPDA and α-ketol of octadecadienoic acid (KODA), but not JA, serve as the mobile signals in *Trichodema*-elicited induced systemic resistance (ISR) [28]. Moreover, while exogenous treatment of maize with MeJA results in increased susceptibility to *C. graminicola* [21], application of 12-OPDA results in increased resistance to this pathogen [27].

JAs signaling activation is initiated upon the perception of JA-isoleucine (JA-Ile), or its functional analog COR produced by a bacterial pathogen *Pseudomonas syringae*, by the receptor protein coronatine insensitive 1 (COI1) [13,17]. COI1 was subsequently identified as the central component of JA signaling module and COR receptor, which upon binding of JAs and COR as ligands directly interacts with the JA repressors, JAZ (jasmonate ZIM-domain) proteins and targets them for degradation [13,29]. JAZs belong to the plant-specific TIFY family, containing a highly conserved TIFY motif (TIF[F/Y]XG) residing within the ZIM domain [30,31,32,33]. Besides Arabidopsis, JAZ gene families were reported in diverse plant species, including maize [34], normally each consisting of multiple members. In the resting state with low levels of active JAs (e.g., JA-Ile) or COR, JA signaling is suppressed by the binding of JAZs to transcription factors (TFs) such as MYC2, which restrains the expression of JA-responsive genes. Elevated levels of JA-Ile or COR triggers the interaction between COI1 and JAZ to form SCF^COI1^/JA-Ile/JAZ ternary complex, resulting in the subsequent ubiquitination and 26S-proteosome-dependent degradation of JAZ protein. The release of MYC2 from JAZ in turn activates the expression of substantial downstream JA-responsive genes [30,31,32]. The discovery of interactions between COI1 and JAZ and, between JAZ and MYC2, led to the proposal of first action module COI1-JAZ-MYC2 in JA signaling pathways [35,36].

Despite the reports of the existence of multiple members of COIs and JAZs in most plant species, the functional redundancy or specificity of individual members remains to be explored [37]. AtJAZ2, for example, was shown to be expressed in guard cells, where it displayed the activity to control COR-regulated stomatal opening [38,39]. Silencing of a flower-specific JAZ in *Nicothiana attenuata* led to the enhanced resistance of flowers against the budworm [40], revealing organ-specific function of this individual JAZ.

MYC2 belongs to the bHLH type TF family and considered as a master TF involved in the regulation of diverse aspects of JA signaling pathways [41]. MYC2 has dual roles in regulating distinct branches in JA signaling pathways, including the positive regulation of wound-induced genes and negative regulation of genes involved in defenses responses to certain pathogens [42]. Intriguingly, MYC2 and its close homologues MYC3 and MYC4 showed redundancy in JA-mediated flowering inhibition in Arabidopsis, likely via regulating transcription of the *Flowering Locus T* gene [43]. Thus, the functional specificity of JA signaling is likely achieved through individual member of JAZs and/or via their interaction with different downstream TFs.

Maize stalk rot caused by *Fusarium graminearum* (teleomorph *Gibberella zeae* Schw. Petch.), also known as Gibberella stalk rot (GSR), is one of the major devastating diseases significantly impacting maize production worldwide. This disease is soil-borne and difficult to control by fungicides and other chemical treatment approaches [44]. Moreover, *F. graminearum* also infects other small grain cereal crops, such as causing Fusarium head blight (FHB), one of the most severe diseases of wheat [45,46]. GSR resistance is a quantitative trait and likely controlled by many QTLs; yet, only a few QTLs or genes have been identified or cloned in maize [44,47,48]. Therefore, the complex molecular mechanisms underlying immunity to GSR in maize remain largely unexplored.

Previous studies have implicated the roles of JAs in resistance or susceptibility to *Fusarium* spp. For instance, it was shown that in a pair of wheat lines with contrasting resistance levels to FHB several genes of JA signaling pathways were upregulated in resistant line [49,50]. Maize JA-deficient *opr7opr8* mutant and *LOX12* mutant, *lox12-1*, a mutant with reduced JA accumulation, were susceptible to Fusarium ear rot caused by *F. verticillioides*, suggesting that JA plays a positive role in the resistance to this pathogen [20]. However, in Arabidopsis infected with *F. graminearum*, JA pathway mutants, including *coi1*, *jar1*, and *opr3*, displayed enhanced resistance [51]. Therefore, the roles of JA in the interactions with *Fusarium* spp. appear to be *Fusarium* species-specific, and may be governed by specific JA-dependent and independent signaling events. These studies prompted a hypothesis that different JA functions, maybe achieved through the activation of functionally diverse COI-JAZ-MYC modules containing distinct individual key components that may even include specific JA ligands.

In this study, we first investigated the effect of exogenous application of MeJA on GSR resistance, and conducted RNA-seq analysis to identify specific members of the families involved in JA biosynthesis and signaling, including JAZ and MYC gene families that may be relevant to maize interactions with *F. graminearum*. Since a set of genes related to JA signaling pathways were differentially regulated by MeJA treatment, we sought to investigate the relevance of JA, 12-OPDA and the components of COI-JAZ-MYC modules to resistance against *F. graminearum* via Y2H screening, protein pull-down assay, and functional analyses using the genetic mutants generated by transposon insertions and CRISPR-cas9 gene editing. We found that COR seems to function as the common ligand mediating the interactions between COI1a and multiple JAZs. Because exogenous MeJA treatment resulted in increased resistance, it was surprising to find that *coi1a* mutant was more resistant, whereas *jaz15* more susceptible, and *opr7opr8* mutant displayed enhanced resistance to GSR. The results demonstrated that ZmCOI1a-ZmJAZ15 signaling module of JA pathway plays an essential role, whereas JA itself might function as a susceptibility factor that facilitates GSR disease progression.

## 2. Results

### 2.1. Exogenous MeJA Treatment Enhanced Maize Resistance to GSR

**Figure 1:**
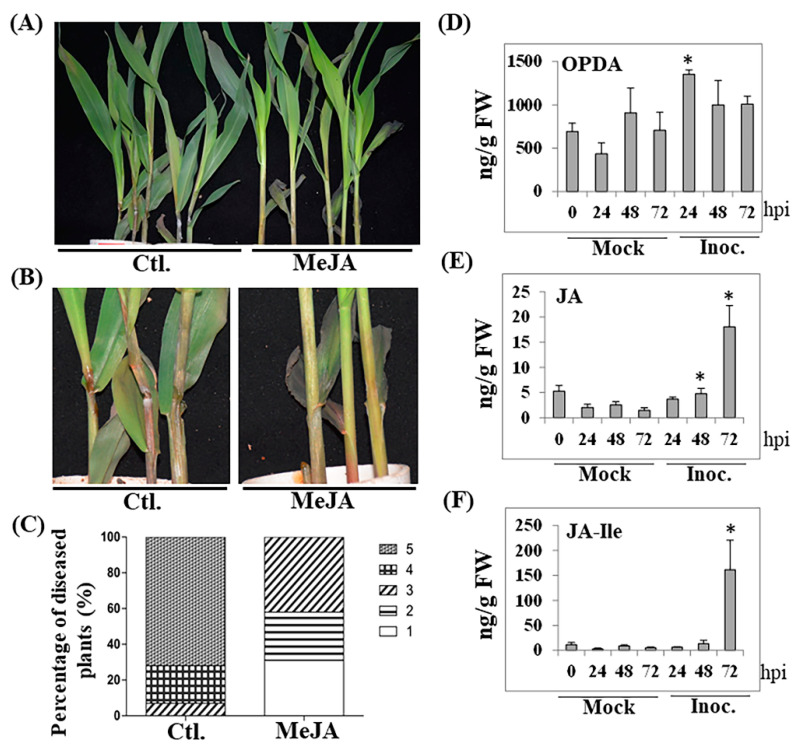
Exogenous MeJA enhanced disease resistance against *F. graminearum*. (**A**) GSR phenotype of whole seedlings of two-week-old B73 spayed with 100 µM of MeJA and that without MeJA treatment, followed by infection with *F. graminearum*. (**B**) the close-up of stems of partial Ctl.- and MeJA-treated seedlings shown in A. (**C**) the quantification of the percentage of diseased plants from A. The percentage of diseased plants was calculated based on the number of plants at each level over total plants. (**D**–**F**) endogenous contents of jasmonic acid and its analogs at different time points (hours post infection, hpi). Mock represents the treatment with H_2_O. The data are the mean of three replicates. * indicates the statistical significance (*p* < 0.01) between mock and inoculation treatments at same time point analyzed by T-test.

To understand whether JAs play a role in maize resistance to GSR, we sprayed 2-week-old seedlings of B73 with MeJA for 12 h, then infected them with *F. graminearum* suspension spores at a concentration of 1.0 × 10^6^/mL. As shown in Figure 1A,B, exogenous application with MeJA enhanced resistance to GSR. While the disease levels of mock treatment ranged from level 3 to level 5, MeJA treatment significantly suppressed disease severity to levels below 3 (Figure 1C). To determine whether endogenous levels of JAs is increased during infection, we quantified major JAs and other oxylipins by LC-MS/MS. Upon infection with *F. graminearum*, the content of the JA precursor, 12-OPDA (Figure 1D), was increased as early as 24 h post infection (hpi), while that of JA (Figure 1E) and JA-Ile (Figure 1F) were reaching higher levels at much later time of 72 hpi.

### 2.2. Genome-Wide Transcription Profiling Identified JA-Biosynthesis and Signaling Genes Activated by MeJA

To identify the specific JA biosynthesis and signaling genes underlying increased resistance of maize to GSR by exogenous MeJA treatment, we performed an RNA-seq analysis using the stems of B73 seedlings following the treatment with MeJA. The raw reads obtained were filtered and subjected to quality control, with the correlation greater than 90% (Pearson *R*^2^ greater than 0.9) among replicates and treatments (Figure S1A), and all data are summarized in Supplementary Table S1. In total, 173 and 112 differentially expressed genes (DEGs) were identified to be upregulated, from 236 DEGs and 112 DEGs ≥ 1.75 fold, from the samples by MeJA treatment for 6 h and 24 h, respectively. Moreover, 28 down-regulated DEGs ≤ 0.5 fold from 34 DEGs ≤ 0.57 fold for 6 h post treatment (hpt), and 39 down-regulated DEGs ≤ 0.5 fold for 24 hpt were identified, respectively (Figure S1B,C; Table S2). Among those genes, 31 genes were upregulated at both time points, while only two were shared among the downregulated genes (Figure S1D).

**Figure 2:**
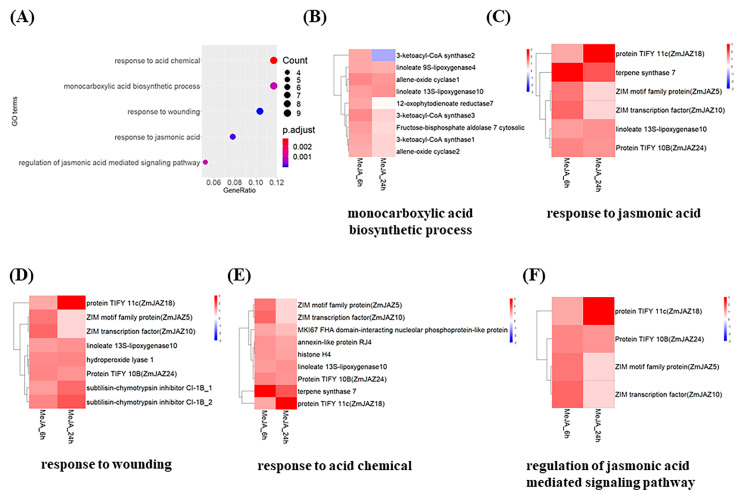
GO enrichment and clustering heat maps on the DEGs detected in MeJA treated stems. (**A**) GO enrichment of all DEGs detected. The color of the dot shows *p*-value of each cluster, and the size of the dot indicates the number of genes enriched. (**B**–**F**) clustering and heatmaps of differentially enriched clusters. The DEGs was selected with a criteria at adjusted *p*-value (*p.adj*) < 0.05 and log2Foldchange > 1 for upregulated genes, while log2Foldchange < −1 for downregulated genes, relative to control. The bar on the side indicates the fold change of corresponding genes over that at 0 h shown on the heatmaps.

GO (Gene Ontology)-enrichment analysis revealed that the DEGs were clustered into five groups, including monocarboxylic acid biosynthetic process, response to acid chemical, response to wounding, response to jasmonic acid and regulation of jasmonic acid mediated signaling pathway (Figure 2A). Among the cluster “monocarboxylic acid biosynthetic process”, *3-ketoacyl-CoA synthase 1* and *3-ketoacyl-CoA synthase 3*, *allene-oxide cyclase 1* and *allene-oxide cyclase 2*, *linoleate 9S-lipoxygease 4* and *linoleate 13S-lipoxygenase 10*, *fructose-bisphosphate aldolase 7 (cytosolic)* were upregulated at both time points, whereas *12-oxophytodienoate reductase 7* (*OPR7*) and *3-ketoacyl-CoA synthase 2* were only upregulated at 6 hpt (Figure 2B). The cluster “response to jasmonic acid” includes *linoleate 13S-lipoxygenase 10, Terpene synthase 7 (TPS7)*, and four *JAZs*, *JAZ5, 10, 18, 24*, among which *TPS7* was up-regulated to a greater level at 6 hpt compared to that at 24 hpt in this cluster (Figure 2C). Moreover, these four *JAZs* were also clustered to “response to wounding,” which also includes *linoleate 13S-lipoxygenase 10, hydroperoxide lyase 1,* and two *substilisin-chymotrypsin inhibitors* (*CI-1B 1 and 2*) (Figure 2D). Besides four JAZs and TPS7, as well as *linoleate 13S-lipoxygenase 10,* mentioned above, *MK167 FHA domain-interaction nucleolar phosphoprotein-like*, *annexin-like protein RJ4,* and *histone H4* were clustered into “response to acid chemical” (Figure 2E). *JAZ5, 10, 18, 24* clustered into “regulation of jasmonic acid mediated signaling pathway” (Figure 2F). Interestingly, jasmonate-biosynthetic and signaling pathways, such as *linoleate 13S-lipoxygenase 10*, and several TIFY-domain containing JAZs proteins (*JAZ5*, *JAZ10*, *JAZ18*) were overlapped in most of these clusters (Figure 2C–E). Taken together, these results suggested that MeJA treatment impacts JA-biosynthetic and signaling pathways, which are highly associated with MeJA-activated resistance to GSR.

To assess how exogenous MeJA impacts the expression pattern of oxylipin pathway genes, the genes involved in JA biosynthetic and other oxylipin pathways were selected and shown in heatmaps (Figure S2). It shows that the majority of 9-*LOX* genes, including *LOX2*, *LOX3*, *LOX4*, and *LOX5* were mainly induced at 24 hpt, particularly *LOX3*, which was induced to much greater levels than other *LOXs* (Figure S2A). Furthermore, most of the *13-LOX* genes, including *LOX7*, *LOX8*, and *LOX9* were slightly induced at both time points, except that of *LOX11* and *LOX13*, which were only induced at 6 hpt. *LOX6* had similar expression pattern as *LOX13*. Members of AOC and AOS gene family were mostly induced at 6 hpt, such as *AOS2*, *AOS3*, *AOC1,* and *AOC2* (Figure S2B,C). However, the expression levels of most *OPR* genes were relatively low, except that of *OPR7*, which was induced to higher level at 6 hpt (Figure S2D). Interestingly, in contrast to *OPRs*, multiple genes involved in SA pathways, such as *PR1*, *PR5*, *HPL*, *PAL*, *PAD4a*, *PAD4b,* and *EDS1* were strongly activated, although *NPR1*, *NPR3,* and *SID2* were not induced (Figure S2D). Taken together, the different responses of distinct pathway genes suggested that SA pathway, 9-LOX pathway and 12-OPDA biosynthesis are more likely to be associated with the enhanced disease resistance to GSR induced by exogenous MeJA treatment, whereas the contribution of JA biosynthesis downstream of AOS/OPR is less evident from this RNA-seq analysis.

### 2.3. Identification of COI-JAZ-bHLH Function Module in Maize Immunity to GSR

Besides JA and other oxylipin biosynthesis genes, another prominent group of genes induced by MeJA included several members of JAZ gene family, suggesting the possible involvement of JAZ in defense against GSR. JAZs family have been previously described in maize using Version 3.0 genomic sequence [34]. Since more recent release of maize genome Ver. 4.0 is much more accurate, we reassessed the composition of JAZ, COI, and bHLH gene families. We identified 6 ZmCOIs and 30 ZmJAZs from the B73 genome Version 4.0 in Gramene (Supplementary Table S3), with 2 and 7 more members, respectively, than previous reports [34,52]. Six ZmCOI genes all contain three exons divided by two introns, while ZmJAZ genes normally possess 1–7 exons (Figure S3A). ZmJAZs are clustered into five distinct groups according to their sequence similarities (Figure S3B). Moreover, the protein sequences of 6 ZmCOIs and 30 ZmJAZs were used as the queries to conduct the chromosomal location analysis. ZmCOI2a, ZmCOI1a, ZmCOI1c, and ZmCOI2b are distributed on first, third, sixth, eighth, and ninth chromosome, respectively, whereas chromosome 8 contains ZmCOI1b and ZmCOI1d. Thirty JAZs genes were localized on ten chromosomes (Figure S4).

Phylogenetic relationship showed that all ZmCOIs form four subgroups, among which ZmCOI2a and ZmCOI2b cluster together with SiCOI2, SbCOI2, and OsCOI1. ZmCOI1a, ZmCOI1b, ZmCOI1c, and ZmCOI1d are grouped with COI1a and COI1b from monocot species, rice, sorghum as well as setaria. Interestingly, AtCOI1 from Arabidopsis and MpCOI1 from a distinct species moss are grouped together to form a group distinct from all other COIs of monocots, while F-box-like proteins from human and Zebrafish form an outgroup from all plant COIs (Figure S5A). All JAZs are clustered into four subgroups, among which most ZmJAZs are closer to SbJAZs and SiJAZs, then OsJAZs, except ZmJAZ10 with OsJAZ28 in group 2 and ZmJAZ22 with OsJAZ5 in group 4. AtJAZs and MpJAZ are normally separated farther away from all JAZs of monocot species (Figure S5B). Moreover, multiple sequence alignment analysis showed that all six ZmCOIs possess conserved transport inhibitor response 1 (TIR1) protein family domain (Figure S6A). All ZmJAZs possess one TIFY domain and one Jas (JA-associated) domain, except ZmJAZ19 which contains a CCT (CONSTANS, CO-like, and TOC1) domain instead of a Jas domain (Figure S6B). The typical TIFY domain contains a “TIFY” motif in most of ZmJAZs, whereas ZmJAZ7, 8, 9, and 19 carry missense residues in their corresponding TIFY.

**Figure 3:**
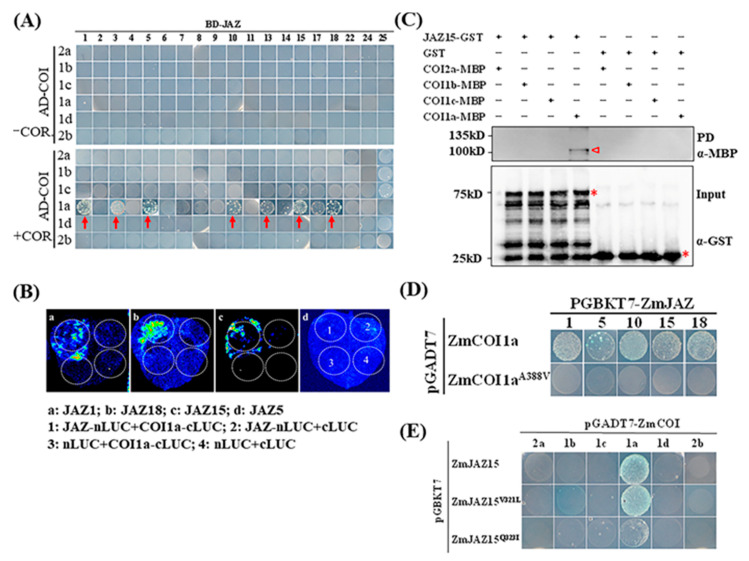
Interaction between COI1a and JAZ15. (**A**) Y2H assay to screen the interaction between all COIs and JAZs proteins. The top panel shows no interaction between any COI and JAZ without COR supplemented in the medium. Bottom panel shows the interaction in the medium containing 20 µM of COR. Red arrows indicate the interaction between COI1a and JAZs. (**B**) Split-luciferase assay confirming the interactions between COI1a and selected JAZs. The panel pictures in a, b and c represent different JAZ members to be tested with COI1a, and d is the schematic diagram for different combination of the vectors. (**C**) Pull-down assay confirming the interaction between COI1a and JAZ15. The assay was conducted using 2 µg COI-MBP and JAZ-GST fusion protein each reaction in the presence of 20 µL GST beads, with 100 nM coronatine. The bands marked in red triangle in top panel shows the signal identified by MBP antibody, and the bands marked in red star in bottom panel shows the input of JAZ-GST fusion protein and GST protein using GST antibody. (**D**) Interaction between different ZmJAZs and ZmCOI1a point mutation in the presence of 20 µM of COR. (**E**) ZmJAZ15 point mutants interacting with ZmCOI1a in the presence of 20 µM of COR.

Although it has been well-known that COI1 could interact with multiple members of JAZs in Arabidopsis [30,31], the specific pairs of COI-JAZ interactors is not known in maize. Thus, we performed a systematic analysis on COI-JAZ interactions in maize. We succeeded in cloning six *ZmCOIs* and 19 out of 30 *ZmJAZs* into the Y2H vector, respectively, and performed the Y2H assays using BD-ZmJAZs as baits. As expected, no interaction was found between any ZmCOI and ZmJAZ without COR in the medium (Figure 3A). On the contrary, several ZmJAZs, including ZmJAZ1, ZmJAZ3, ZmJAZ5, ZmJAZ10, ZmJAZ13, ZmJAZ15, and ZmJAZ18 were found to interact with ZmCOI1a, but not other ZmCOIs, in the presence of COR at a concentration of 20 µM. To confirm the interaction between ZmJAZs and ZmCOIs, we performed the split-luciferase assay by constructing the selected ZmJAZs and ZmCOIs into the C-terminus and N-terminus of luciferase, and co-infiltrated them using Agrobacteria into *Nicothiana benthamiana* leaves. It was found that ZmJAZ1, ZmJAZ15, and ZmJAZ18 all interacted with ZmCOI1a, whereas no interaction between ZmJAZ5 and ZmCOIs was detected (Figure 3B). The interaction between ZmJAZ15 and ZmCOI1a was also confirmed by GST pull-down (PD) assay (Figure 3C), suggesting that ZmCOI1a likely functions as an essential component in maize JA signaling complex.

Similar to Arabidopsis COI1, ZmCOIs also contain F-box-like and TIR1 protein domains (Figure S7A), while TIFY and Jas domains consisting of Degron and α-loop subdomains are highly conserved in maize JAZs, in which ZmJAZ19 lacks a canonical Jas domain compared to other members (Figure S7B). To examine the conserved functional binding sites for ZmCOI1a-ZmJAZ15, we first created point mutation in one of the leucine-rich repeat motif of ZmCOI1a, by changing residue alanine (A) to valine (V) at position 388 of amino acid sequences. This amino acid was found to be essential for COI1 interaction with JAZ in Arabidopsis [53]. Compared to wild type ZmCOI1a, ZmCOI1a^A388V^ (Figure S8) failed to interact with any member of JAZs (Figure 3D). To further confirm the function of conserved residues in COI-JAZ interactions, two point mutations conserved in the CCT domain were generated, including ZmJAZ15^V321L^ and ZmJAZ15^Q323I^, and Y2H assay was performed. While ZmJAZ15^Q323I^ showed slightly reduced interaction with ZmCOI1a, ZmJAZ15^V321L^ mutation did not impact the interaction of ZmJAZ15 with ZmCOI1a, when compared to WT (Figure 3E).

**Figure 4:**
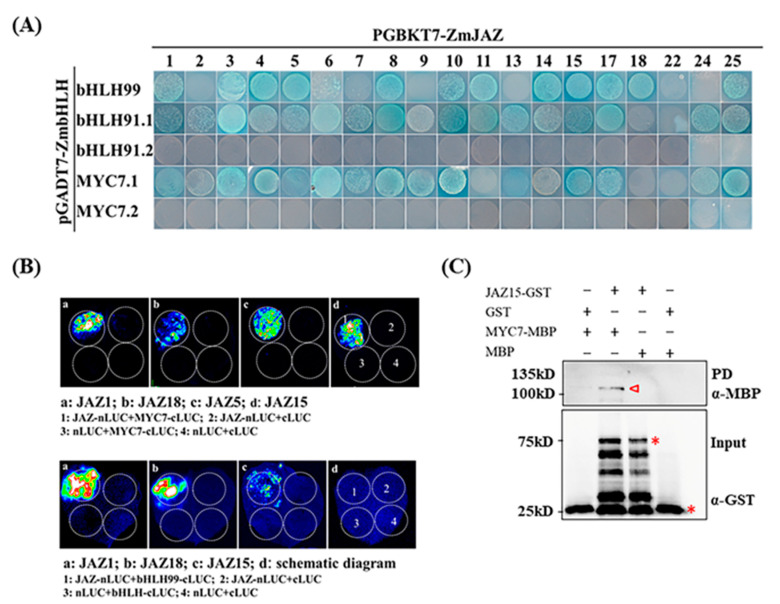
Identification of interactions between JAZs and bHLHs. (**A**) Y2H assay to screen the interaction between all JAZs and bHLH99, bHLH91 and MYC7 proteins. bHLH91.1 represents the N-terminus of bHLH91 while bHLH91.2 represents C-terminus. MYC7.1 represents the N-terminus of MYC7 and MYC7.2 for C-terminus. (**B**) Split-luciferase assay confirming the interactions between selected JAZs and MYC7 (top panels) and bHLH99 (bottom panels). Panel pictures in a, b and c represent different JAZ members to be tested with either MYC7 or bHLH99, and d is the schematic diagram for different combination of the vectors. (**C**), Pull-down assay confirming the interaction between JAZ15 and MYC7. The assay was conducted using 2 µg COI1-MBP and JAZ-GST fusion protein each reaction in the presence of 20 µL GST beads. The bands marked in red triangle in top panel shows the signal identified by MBP antibody, and the bands marked in red star in bottom panel shows the input of JAZ-GST fusion protein and GST protein using GST antibody.

It has been reported that there are 208 members of bHLHs family in maize [54], making it impractical to test the entire family. To investigate whether the JAZ-bHLH module also functions in JA signaling pathway in maize, we cloned three maize representative bHLHs, bHLH99, bHLH91, and MYC7, which are the closest homologs to MYC2, MYC3, and MYC4 in Arabidopsis (Supplementary Table S4). The interactions between selected ZmJAZs and ZmbHLHs were investigated by Y2H assay. As shown in Figure 4A, most of the ZmJAZs interact with bHLH99, except ZmJAZ2, ZmJAZ6, ZmJAZ7, ZmJAZ9, ZmJAZ13, ZmJAZ22, and ZmJAZ24. Since both full-length of bHLH91 and MYC7 appear to have self-toxic activity to yeast cells (data not shown), we constructed the N-terminal (bHLH91.1) and C-terminal (bHLH91.2) protein derivatives of both bHLH91 and MYC7 (MYC7.1 and MYC7.2). Both bHLH91.1 and MYC7.1 contain JID (JAZ-interaction domain) domains and activation domains, and thus are sufficient for the interaction with most of the ZmJAZs, except ZmJAZ18 and ZmJAZ22 for bHLH91 and ZmJAZ11, ZmJAZ18, and ZmJAZ22 for MYC7. bHLH91.2 and MYC7.2 alone did not interact with any ZmJAZs (Figure 4A). Furthermore, the interactions between selected ZmJAZs, including ZmJAZ1, ZmJZ18, ZmJAZ5, and ZmJAZ15, with both MYC7 and bHLH91 were verified by split-luciferase assay (Figure 4B). We further confirmed the interaction between ZmJAZ15 and MYC7 by GST pull-down assay (Figure 4C).

To understand if increased concentration of COR affects the interactions between ZmCOIs and ZmJAZs, we first performed Y2H assay for ZmCOI1a and ZmJAZ7, ZmJAZ18, ZmJAZ15, and ZmJAZ24 by adding COR in the medium (Figure S9). It showed that ZmCOI1a still interacted with ZmJAZ15 and ZmJAZ18 when COR concentration was increased to 100 µM, as that in the medium containing 20 µM COR, whereas no interaction was found between ZmCOI1a and ZmJAZ7, suggesting that the interaction between ZmCOI1a and ZmJAZs is likely ligand concentration-dependent.

### 2.4. Functions of COI1-JAZ Action Module in Maize Immunity to GSR

Given the essential role of ZmCOI1a in forming protein–protein complexes, we examined the function of ZmCOI1a in maize interactions with *F. graminearum*, by deploying CRISPR-Cas9-based gene editing approach. We constructed the plasmid harboring two sgRNA targeting sites in the two exons of *ZmCOI1a* (Figure S10A), one of which is F-box, and the chimeric plasmid was delivered into the inbred KN5585 embryos via *Agrobacterium tumefaciens* transfection. T0 plantlets (288 in total) were obtained, 217 being positive (75.3%) in 18 transgenic events. However, only three allelic lines, M180815A010a (06086), M180815A014a (06087), and M180815A026a (08166), were available for phenotypic analysis, because of our inability to produce sufficient seeds caused by the flowering mismatch between male and female organs. Sanger sequencing confirmed that the mutant allele 06086 contains a single nt insertion at target 1 (A) and two nt deletion (TC) at target 2 (Figure S10B), whereas only 14 nt deletion at target 2 was detected in the allele 06087 (Figure S10C). The allele 08166 has 15 nt insertion at target 1 and one nt deletion at target 2 (Figure S10D). To further confirm the deficiency of *zmcoi1a* mutants in JA signaling, we deployed the root inhibition assay to compare the root length of WT and mutants in response to the treatment of exogenous JA. The mutants 06086 and 06087 became less sensitive to JA, with 59.3% and 57% reduction, respectively, compared to that of WT treated with 25 uM JA, with 69.9% reduction of root length (Figure S11). Unfortunately, we were not able to examine line 08166 in root assay because of insufficient seeds.

Since ZmJAZ15 showed strong and stable interaction with ZmCOI1a evidenced by Y2H, PD, and split-Luciferase assays, we decided to construct the KO lines of *ZmJAZ15* using CRISPR-cas9 genome editing techniques. Two gRNAs target sites were designed to generate DSBs in F-BOX motif (blue bar inside first exon) and on the CCT domain of ZmJAZ15, which resulted in three T0 KO homozygous lines. Sanger sequencing confirmed that the mutant allele M180980A003A (08591) contains 8 nt deletion at target 1 (A), whereas a single nt “T” insertion was detected in the alleles M180980A001A (08592), respectively (Figure S12).

**Figure 5:**
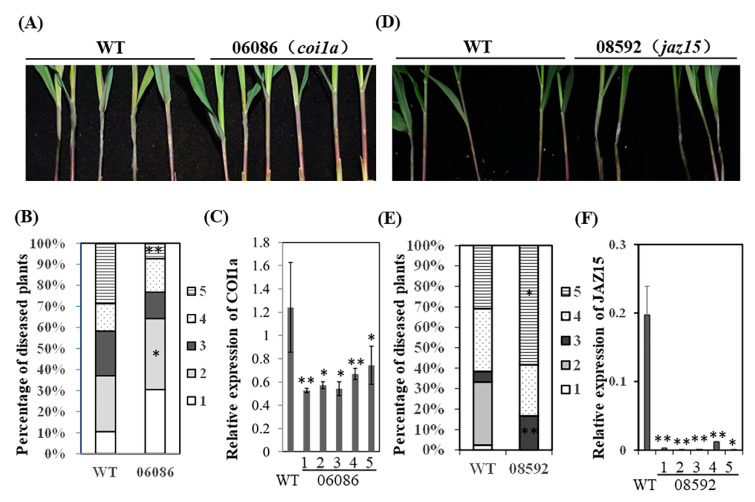
CRISPR-cas9 KO *coi1a* mutant is more resistant, whereas *jaz15* mutant more susceptible to GSR. (**A**) GSR phenotypes of *coi1a* KO line and WT on 4 dpi. Only part of seedlings were shown in the picture. (**B**) the disease index distribution of each line. The disease index was recorded using level 1~5, in which 1 indicates the most resistant and 5 the most susceptible. The percentage of diseased plants was calculated based on the number of plants at each level over total plants. The star on the side of each level indicates the significance of difference of disease index in mutant over WT. (**C**) the expression levels of *COI1a* in different seedlings of line 06086. (**D**) GSR phenotypes of *jaz15* KO line and WT on 4 dpi. Only part of seedlings were shown in the picture. (**E**) the disease index distribution of each line. The disease index was recorded using level 1~5, in which 1 indicates the most resistant and 5 the most susceptible. The percentage of diseased plants was calculated based on the number of plants at each level over total plants. The star on the side of each level indicates the significance of difference of disease index in mutant over WT. (**F**) the expression levels of *JAZ15* in different seedlings of line 08592. The number under the bar on X-axis corresponds individual seedling shown in the picture in **A**. WT represents the average expression level from multiple seedlings. At least ten seedlings per line were tested for the phenotype and the experiments were replicated for three times with similar results. The statistical analysis was performed by T-test, and * and ** on the bars indicate significant differences between mutants and wild-type at *p* < 0.05 and *p* < 0.01 level, respectively.

To identify the roles of *ZmCOI1a* and *ZmJAZ15* in resistance to GSR, we infected WT and CRISPR-cas9 knock-out mutants with *F. graminearum* and scored the disease severity at 4 dpi. The data showed that the KO allele 06086 of *zmcoi1a* became more resistant to GSR (Figure 5A), as demonstrated by the significantly more plants displaying level 2 and less level 5 in the mutant compared to WT (Figure 5B). qRT-PCR analysis showed that the expression levels of *ZmCOI1a* in all individual seedlings of these three alleles examined were suppressed, with the reduction ranging from 25% to 75% (Figure 5C). Reversely, the CRISPR-cas9 KO allele 08592 of *ZmJAZ15* was more susceptible to GSR (Figure 5D), with all seedings displaying disease level above 3, in which more diseased plants were found to be level 5, compared to that of WT, for which all seedling disease levels ranged between level 1 and 5 (Figure 5E). Furthermore, the very low gene expression of *ZmJAZ15* was detected in each individuals of allele 08592 (Figure 5F). Therefore, the data suggested that *ZmCOI1a* likely played a positive role, whereas *ZmJAZ15* played a negative role in the resistance to GSR.

### 2.5. opr7opr8 Double Mutants Are More Resistant to GSR

**Figure 6:**
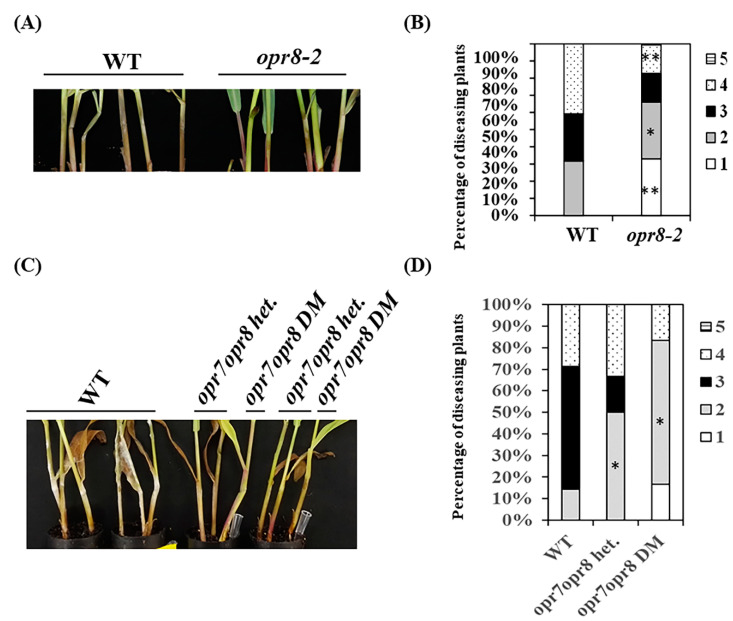
*opr7opr8* mutants are more resistant to *F. graminearum*. (**A**) a representative picture shows the disease symptoms of *opr8-2* mutant vs. WT at 3 dpi; (**B**) the disease levels of WT and *opr8-2* mutants recorded at 3 dpi; (**C**) a representative picture shows the disease symptoms of *opr7-5opr8-2* DM, *opr7-5opr8-2* heterozygous and WT at 4 dpi; (**D**) the disease levels of WT, *opr7-5opr8-2* heterozygous and *opr7-5opr8-2* DM recorded at 3 dpi. The percentage of diseased plants was calculated based on the number of plants at each level over total plants. The experiments were replicated three times with similar results and the representative data are shown. * and ** on the bars indicate significant differences between mutants and wild-type at *p* < 0.05 and *p* < 0.01 level, respectively, analyzed by the two-way ANOVA.

To provide additional line of evidence for the role of JAs in maize interactions with *F. graminearum*, we examined GSR disease symptoms of maize JA-deficient *opr7-5opr8-2* double mutants (DM) and *opr8-2* single mutant, which is still able to produce JA but at lower levels [18]. Single *opr8-2* showed enhanced resistance to GSR (Figure 6A,B). Furthermore, *opr7-5opr8-2* DM displayed increased resistance to this disease (Figure 6C,D), suggesting that JA promotes susceptibility rather than defense against this pathogen in maize stems.

## 3. Discussion

### 3.1. Maize Susceptibility to F. graminearum Is Promoted by JA and COI1a

Despite the major losses occurring on maize caused by *F. graminearum* infection, our understanding of the contribution of specific defense hormones to maize resistance or susceptibility to this pathogen is limited. In this study, we elucidated the relevance of JA to maize response to this hemibiotrophic pathogen. For this, we have utilized both pharmacological approach and genetic mutants of JA biosynthesis and signaling pathways, and comprehensively characterized the protein–protein interactions between the members of COIs, JAZs, and bHLHs families of maize.

As the central component of JA signaling pathway, COIs have been extensively reported for their essential role in diverse biological processes including development and growth, defense response against pathogens and insects, and tolerance to abiotic stress [16,29,55,56]. COI1 functions together with CUL1, Rbx1, and Skp1-like proteins as a receptor complex SCF^COI^ E3 ligase [29,57], binding JAs ligands to trigger downstream biological processes. For instance, a large set of genes induced by exogenous JA treatment are no longer inducible in Arabidopsis *coi1* mutant [58]. Moreover, loss-of-function of tomato COI homolog caused severe defects in JA-promoted pollen viability, trichome formation, and defense response against spider mites [59]. Thus, COIs exert their function as a core receptor to perceive and transduce external signal, through COI-JAZ-MYC2 action module to trigger downstream biological responses.

Accumulating evidence suggested that, in addition to mediating defenses against necrotrophic pathogens and chewing insects, certain pathogens could arrogate JA signaling components to promote disease or parasitism [60,61]. In Arabidopsis, for example, *coi1* mutants showed enhanced resistance to wilt disease caused by *F. oxysporum* [62]. Moreover, it has been reported that COI1 could function in Arabidopsis roots to transmit the root to shoot signaling that is independent of JA-Ile ligand resulting in wilt disease symptoms caused by *Verticillium longisporum* [63]. It has been widely accepted that COR, avirulence factor produced by some bacterial pathogens, mimics JA-Ile, both functioning as active ligands for COI-JAZs receptor complex to trigger JA signaling, confirmed by structural, biochemical, and pharmacological means [13,29,57]. The activation of JA signaling by COR resulted in subsequent suppression of SA-mediated immunity to a hemibiotrophic pathogen *Pseudomonas syringae*, eventually leading to disease symptoms in plants [64,65]. Similarly, *P. syringae* effector AvrRpm1/AvrRpt2 promoted bacterial growth and host chlorosis via COI1, likely through the suppression of SA-mediated immune response [66]. In this study, COI1a was identified as a key JA signaling component of COI-JAZ-MYC7 module in maize, as demonstrated by in vitro Y2H screening and pull-down assay, as well as in vivo Split-Luc assay. While exogenous MeJA enhanced maize resistance to GSR, unexpectedly, *coi1a* CRIPSR-cas9 KO mutant and *opr7/opr8* DM were more resistant to GSR, whereas the mutation of the JAZ repressor, *ZmJAZ15*, resulted in increased susceptibility to this pathogen. These findings further supported the idea that JA and COI1a likely function in promoting disease susceptibility of GSR.

**Figure 7:**
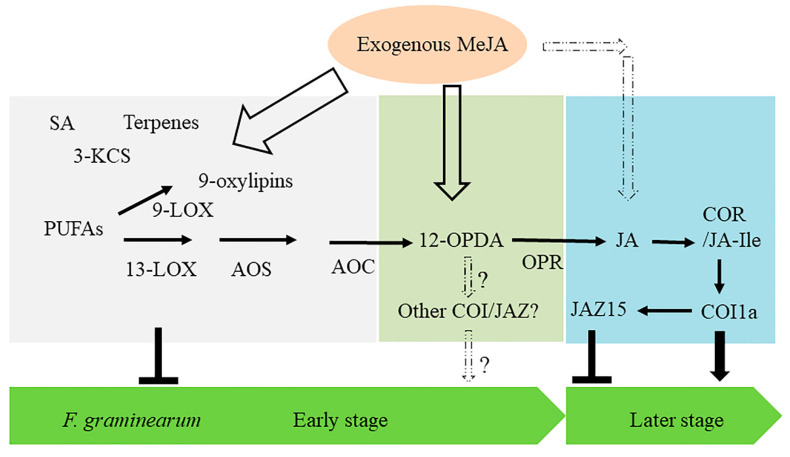
Working model of ZmCOIa-ZmJAZ15 function module in the immunity to GSR. The filled arrows indicate the activation of signaling components by MeJA treatment in JA pathways, and the dashed open arrows indicate to-be-verified activation by exogenous MeJA treatment. The wider the arrows, the stronger the effects. The solid black arrows and bars represent the positive and negative role in disease development, respectively.

Despite that we noted the enhanced resistance of maize stems to GSR by exogenously applied MeJA, this effect is likely due to the induction of oxylipins other than JA. MeJA-inducible resistance to GSR is likely independent of COI1a, or due to the yet unknown JA-independent defense pathways induced by MeJA, such as 9-LOX signaling pathways as shown recently for a 9-oxylipin alfa-ketol KODA of gamma-ketols [27,28]. Another candidate molecule is probably 12-OPDA that was recently shown to induce resistance to other hemibiotrophic pathogens [27] (Figure 7).

### 3.2. Screening of COI-JAZ and MYC Protein Complexes Identified ZmCOI1a-ZmJAZ15 as an Essential Module in Maize-Fusarium Interaction

Using CRISPR-cas9 KO mutants, we showed that COI1a promotes susceptibility to *F. graminearum*. A study using association population mapping revealed that two sunflower homologs of COI1 are associated with *Sclerotia* stalk rot resistance [60]. Thus, it appears that the role of COI1 and JA in general is strongly dependent on the pathogen species or more specifically on their life styles; *Sclerotinia* pathogen is a necrotroph, defenses to which rely on JA, whereas *F. graminearum* has a hemi-biotrophic life style, the defenses to which are typically associated with SA.

The first COI1-JA-JAZs receptor module was identified in Arabidopsis, since the discovery of JAZ proteins [30,31,32]. Although there are numerous studies on the characterization and functional analysis of COI1 or JAZs solely [34,52,53,67,68], this study provided insightful information about the complexity and binding specificities of COI-JA-JAZ action module in maize. One possibility for COIs’ functional redundancy involved in GSR immunity is due to the diversity and complexity of COIs homologous genes in maize. It has been widely known that Arabidopsis possesses a single copy of the COI1 gene, whereas monocot species contain multiple copies of COI1 or COI1-like genes in their genomes. For instance, wheat has two COI1s, and *Brachypodium distachyon* has at least three COI1 homologs that are highly similar to Arabidopsis COI1 [69,70]. Moreover, three COI1 homologs, OsCOI1a, OsCOI1b, and OsCOI2, have been identified in rice, among which only OsCOI1a and OsCOI1b, but not OsCOI2, were able to complement the JA-deficient phenotype in Arabidopsis *coi1* mutants [68], indicating that the diversity of COIs in monocots is more complex compared to dicot species [71]. Such diversification of COIs and JAZs family members may help tightly regulate the diversity of biological processes controlled by JA signaling. Interestingly, another recent report showed that maize genome contains four COI1 homologs, COI1a, COI1b, COI1c, and COI2, among which ZmCOI1s (not ZmCOI2s) could restore the male fertility and activate defense response to fungal pathogens in the *coi1* mutant of Arabidopsis [52]. By using more recently released maize genome data and comprehensive functional approaches, our present study identified six members of the COIs family and 30 members of JAZs in maize, further substantiating the functional diversity and potential redundancy of COI-JAZ action modules in maize.

Using Y2H assay, we could only detect the interactions between ZmCOI1a and several ZmJAZs in the presence of COR ligands, but not JA-Ile in yeast system (data not shown), which needs to be further tested in in vivo. Interestingly, a comparison study on the binding affinity for COR between AtCOI1 and OsCOI1 clearly showed that AtCOI1 possessed a binding constant (Kd) value of 0.96 µM and OsCOI1 of 7.63 µM, in an ITC assay in vivo, and 0.1 µM for the former and 0.52 µM for the latter using in vitro ITC assay, respectively [72], suggesting the differential preference of COI1 receptor by different host species perceiving same ligand molecules.

Given the negative role of COI1a in immunity to GSR (Figure 7), it is attempting to speculate that COI1a maybe highjacked by *F. graminearum* in a manner similar to the study, which showed that certain strains of *F. oxysporum* could utilize jasmonates as effectors to boost its pathogenicity in tomato plant [69]. It remains to be explored, however, if *F. graminearum* produces any jasmonates or other oxylipins such as JA-Ile, 12-OPDA, or 12-OPDA-Ile that potentially could activate JA signaling module, similar to that by COR produced by *P. syringae*.

### 3.3. Ligand-Specific Activation of JA Signaling Pathways in Maize Interactions with F. graminearum

Besides COR and JA-Ile, several JA-derivates or analogs are increasingly reported to function either as ligands or abettors in JA signaling pathway. For instance, OPDA-Ile [11] and 12-OH-JA-Ile [73] were also identified to require COI1 for signaling, respectively, while two isomeric forms of the JA-Ile precursor dinor-OPDA (dinor-*cis*-OPDA and dinor-*iso*-OPDA) seem to be unique ligands for *Marchantia polymorpha* MpCOI1 [74]. Moreover, another compound, inositol pentakisphosphate, was also identified as an accessory component of JA-Ile to facilitate the formation of COI1-JAZ receptor complex [56], unveiling the additional layers in the complexity of ligand-receptor specificity in JA signaling. It has been previously reported that 12-OPDA, not JA, serves as a signal to enhance maize resistance to aphids, indicating the JA-independent role of 12-OPDA [26]. More recently, 12-OPDA has also been shown to function as a long-distance signal to activate *Trichoderma*-induced ISR against fungal pathogen in maize [28], and in defense against herbivory insects [75].

Because our combinative assays using Y2H screen, Split-Luciferase and PD assay identified only one of the six maize COI1 homologues, COI1a, as an interactor with most of the JAZ proteins in the presence of COR ligand, we hypothesized that other COIs may require a different jasmonate ligand such as 12-OPDA.

Given that *opr7-5opr8-2* double mutation also impacted the production of 12-OPDA [18], it is reasonable to propose that 12-OPDA may also trigger the formation of different action module rather than COI1a-JAZ15, which will further support the distinct yet unique signaling mechanisms of JA signaling and biosynthetic pathway components in defense response against GSR. More specifically, 12-OPDA decreased after 2 and 3 days following the inoculation with *F. graminearum* in this study, it is reasonable to speculate that 12-OPDA-mediatd additional COI-JAZ module may promote the GSR disease susceptibility (Figure 7). Further detailed analyses will be required in the future to elucidate whether 12-OPDA could serve as an effective ligand for at least some of the COI1s in maize. Furthermore, extensive investigation on 12-OPDA binding affinity of the receptor will enhance our understanding of the dynamics and complexity of JA signaling pathways in monocots than dicots.

### 3.4. Other Components Possibly Involved in Defense Activated by MeJA-Treatment

In our RNA-seq analysis, besides JA-biosynthetic and signaling pathway genes, multiple genes involved in other biological and biochemical processes, such as *9-LOXs*, *TPS7*, *substilisin-chymotrypsin inhibitor C1* (*SCI-C1*), *3-KCS* and *fructose-bisphosphotase aldolase 7 cytosolic* (*FBA7c*) and SA pathway genes are also induced by MeJA treatment at early time points, suggesting the possible involvement of these components in MeJA-activated immunity to GSR (Figure 7).

Among above genes, TPSs and terpenes have been broadly acknowledged for their roles in various defense-related processes [76]. TPSs belong to an enzyme family that catalyze the formation of a series of secondary metabolites, initiated from C5-unit isopentenyl diphosphate (IPP), consisting of hemiterpenes, monoterpenes, sesquiterpenes, and diterpenes, some of which are various volatile compounds [77]. Accumulating evidence also showed that JA could stimulate terpene production to shape plant interactions with adverse stimuli, particularly pathogens and insects [78,79,80]. Intriguingly, MYC2, the key transcription factor of JA signaling pathway in Arabidopsis, has been shown to integrate JA signaling with GA signaling component DELLA via binding to the promoter of *TPS11* and *TPS21*, to regulate their gene expression, thus the defense against insects [81]. Furthermore, *TPS10* from maize was reported to be induced by herbivory damage [82], indicating its potential role involved in defense against broad-spectrum biotic stresses.

Moreover, multiple genes in SA pathways, such as *PR1*, *PR5*, *PAL1,* and *PAD4,* were also upregulated, especially at early stage in response to infection with *F. graminearum*, implicating their potential roles in suppression GSR disease development (Figure 7). Additionally, another group of genes, *3-KCS* was also found to be upregulated during the interaction between maize and GSR. *3-KCS* was previously reported to function in plant fatty acid elongation and is required for synthesis of epicuticular waxes [83], which was believed to be associated with barley resistance against *Magnaporthe oryzae* [84]; however, their involvement in maize defense against *F. greaminearum* has not been investigated. The crosstalk between SA and JA has been broadly reported to function differentially in regulating biotrophic and necrotrophic phases of pathogens [85]. Taken together, all these information warrants future examination of the functions of *TPS7*, SA, and *3-KCS*, as well as the crosstalk between different phytohormonal and metabolism pathways in MeJA-activated maize immunity to GSR (Figure 7).

## 4. Materials and Methods

### 4.1. Plant Materials, MeJA Treatment, and GSR Seedling Assay

Maize inbred lines B73 and KN5585 were propagated and maintained regularly. CRISPR-cas9 knockout (KO) mutant materials were generated in KN5585 background and propagated in greenhouses in Xishuangbanna, Yunnan Province, China, by WIMI Biotechnology Co., Ltd. (Changzhou, China), and T2 seeds were used for phenotype analysis. Mutator insertion mutants, *opr7*, *opr8,* and *opr7-5opr8-2* double mutant, were created as described by Yan et al. [18]. The seeds were sown in the soil in long pots made with PVC cylinders (5 cm in diameter and 20 cm in depth) and grown under 14 h light/10 h dark conditions for 2 weeks. The seedlings were then sprayed with 100 µM of MeJA dissolved in 0.1% acetone, and stems were used for GSR phenotyping at 3~4 days post infection (dpi), or harvested at designated time points, frozen in liquid N2 immediately and stored in −80 °C until use for RNA-seq analysis or gene expression study. The control plants received the same amount of 0.1% acetone. To prevent the evaporation of MeJA volatiles, the trays containing treated seedlings were covered immediately with PressIn saran wrap and kept at the same conditions. Each treatment per time points consists of at least six seedlings for RNA-seq and ten for GSR phenotyping. The experiments were conducted in at least three biological replicates with constant results obtained. GSR assay was conducted as described previously by Sun et al. [86] with slight modification. Twenty-four hours after treatment with MeJA, the stems were infected with *F. graminearum* suspension spores at a concentration of 1.0 × 10^6^/mL in 0.001% Tween-20. GSR symptoms were scored at 3~4 dpi.

### 4.2. RNA-seq and qRT-PCR

RNA-seq library was constructed using Illumina TruSeq RNA library prep kit v2 and RNA sequencing was conducted using Illumina HiSeq 2000 (Illumina Inc., San Diego, CA, USA) by BerryGenomics company (Beijing, China). Paired end RNA sequence reads were generated, cleaned, and aligned to B73 reference genome (RefGen_V4) using Hisat2. The gene expression value was normalized as gene counts. The differently expressed genes (DEGs) were produced with the threshold of a false-discovery rate (FDR) < 0.05 by the DESeq software Packages (bioconductor.org/), selected with a criteria at adjusted *p*-value (*p.adj*) < 0.05 and log2Foldchange > 1 for upregulated genes, while log2Foldchange < −1 for downregulated genes, relative to control. Three independent biological replicates were conducted with each replicate containing six seedlings. Total RNA was isolated from maize stems using TRIzol reagent according to the manufacturer’s protocol (Invitrogen), and cDNA was synthesized using HiScript QRT SuperMIX for quantitative PCR (R233-01, Vazyme). Quantitative RT–PCR analysis was performed using the AceQ qPCR RT SYBR Green Master Mix (Q212-01, Vazyme) in a Bio-Rad real-time instrument (CFX96). The PCR program consisted of an initial denaturation step at 95 °C for 5 min, followed by 40 cycles of 95 °C for 30 s, 60 °C for 30 s. All quantitative RT–PCR reactions were performed with three biological replicates for each sample. The transcript level of a *β-Tublin 4* gene (Zm00001d013612) was used as internal normalization. The experiments were conducted at least in three biological replicates. qRT-PCR primers were listed in Table S5.

### 4.3. Yeast-Two-Hybrid (Y2H) Assay

Y2H assays were performed using the Matchmaker GAL4 two-hybrid systems (Clontech, Mountain View, CA, USA). The baits ZmJAZs were fused with BD domain in the pGBKT7 vector and preys ZmCOIs or ZmbHLHs with AD domain in pGADT7 vector, which were co-transformed into yeast cells by PEG/LiAc solution-mediated transformation. Co-transformants were grown on 2×YPDA liquid medium, and incubated at 30 °C by shaking at 200 rpm for 12–16 h. Yeast synthetic dropout medium lacking Leu and Trp (DDO) was used to test the efficiency of co-transformation, while selective media lacking Ade, His, Leu, and Trp (QDO) was used to test the protein interactions. When verifying the interaction between ZmCOIs and ZmJAZs, 20 µM coronatine was added to the QDO plate. The empty pGADT7-T vector was co-transformed with pGBKT7-53 or pGBKT7-Lam in parallel as a positive and negative control. The yeast cells were incubated at 30 °C for 3d and the protein interactions were checked on QDO medium. Primers used for the Y2H are listed in Table S5.

### 4.4. Split-Luciferase Assay

ZmJAZs were fused with nLUC individually in pCAMBIA1300-nLUC vector, while ZmCOIs or ZmbHLHs fused with cLUC were individually cloned into pCAMBIA1300-cLUC vector. The plasmids were subsequently transformed into *Agrobacerium tumefaciens* strain GV3101. The bacteria containing the recombinant vector were grown on LB medium with 50 mg/L kanamycin and 50 mg/L rifampicin for 24 h, then collected by centrifugation, and resuspended in infiltration solution (10 mM MES, 10 mM MgCl_2_ and 200 μM acetosyringone) by adjusting the concentration to OD_600_ = 1. When verifying the interaction between ZmCOIs and ZmJAZs, 200 nM coronatine was supplemented into infiltration solution. Equal concentration and volume of Agrobacterium strains were mixed with helper component proteinase (Hcpro) and co-infiltrated into young but fully expanded *Nicotiana benthamiana* leaves using a needleless syringe. The infiltrated plants were immediately placed to a complete dark at 23 °C for 48 h before they were incubated under a condition of 16-h light/8-h dark for 16 h. The plants were then spayed with 1 mM luciferin (MDBio, Qingdao, Shandong, China) containing 0.01% Triton X-100, and the leaves were kept in the dark for 10 min to quench the fluorescence. LCI images were captured with a low-light cooled CCD imaging apparatus (Tanon, China). Primers used for the vector construction are shown in Table S5.

### 4.5. Pull-Down Assay

ZmJAZs fused to glutathione-S- transferase (GST) in vector pGEX-6P-1 were purified using BeyoGoldTM GST-tag Purification Resin (MDBio, Qingdao, Shandong, China). ZmCOIs and ZmMYC7 were cloned to pMAL-C2 and purified using amylose resin (NEB), respectively. Approximately 2 μg of proteins each were mixed in 1 mL of pull-down buffer (50 mM Tris-HCl pH 7.5, 200 mM NaCl, 0.5% TritonX-100, 0.5 mM β-mercaptoethanol), and incubated at room temperature for 2 h with rotation, then 10 uL of GST beads were washed twice using the pull-down buffer, then added to each reaction and incubated under the same condition for another 2 h. GST beads alone were used as a control. The GST beads were spun down, washed five times with the pull-down buffer, resuspended in 5 × SDS buffer, and resolved on 12% SDS-PAGE gel and detected by anti-MBP (TransGen) antibody. The loading control of GST proteins was verified using anti-GST antibody. When examining the interaction between ZmCOIs and ZmJAZs, COR at 200 nM was added to the pull-down buffer. The pull-down assays were repeated at least three times with similar results and the representative results were presented.

### 4.6. Measurement of JA and Its Derivates Oxylipins

Endogenous levels of JA and its derivative compounds in B73 stems upon *F. graminearum* infection were quantified by comparing the levels of endogenous metabolites to isotopically labeled standards from Sigma-Aldrich (St. Louis, MO, 713 USA), Cayman Chemical (Ann Arbor, MI, USA), and Larodan AB (Solna, Sweden), using liquid chromatography tandem mass spectrometry (LC-MS/MS) according to the description by Wang et al. [28]. Briefly, 100 mg of stem tissue was finely ground using liquid nitrogen and mixed with 500 μL extraction buffer (1-propanol/water/HCl (2:1:0.002 vol/vol/vol)) containing 10 μL of 5 μM isotopically labeled internal standards. The samples were agitated, evaporated using N2 gas stream, then 90 uL of supernatant was transferred into autosampler vials with glass inserts, with a 15 μL aliquot being injected into an API 3200 (Sciex, Framingham, MA, USA) LC-MS/MS using electrospray ionization in negative mode with multiple reaction mentoring (MRM). The chromatography was performed with an Ascentis Express C-18 Column (3 cm × 2.1 mm, 2.7 μm) (Sigma-Aldrich, St. Louis, MO, USA). The mobile phase was set at 400 mL/ min consisting of Solution A (0.02% acetic acid in water) and Solution B (0.02% acetic acid in acetonitrile) with a gradient consisting of (time in min—%B): 0.5–10%, 1.0–20%, 21.0–70%, 24.6–100%, 24.8–10%, 29–stop. The peaks and retention times of each sample were integrated and analyzed by comparing that with standards using appropriate response factors.

### 4.7. Mutagenesis of ZmCOI1a and ZmJAZ15 with CRISPR/Cas9-Based Gene Editing and Transformation

To verify the function of COI1a and JAZ15 in the immunity to GSR, we employed the CRISPR-cas9 techniques to knock-out the key sites in the sequences of *COI1a* and *JAZ15*, respectively, which were accomplished by WIMI Biotechnology Co., Ltd. (Changzhou, China), according to the previous reports by Liu et al. [87] and Xing et al. [88] with some modifications. Two RNA sequences (CCCCAACCTAAGTGTTCTCGAGG and ACATGGACGACCCGCGAGACCGG) targeting the first exon flanking the F-box and the second exon of *COI1a* respectively, were designed according to the online protocol CRISPR-P 2.0 (http://crispr.hzau.edu.cn/CRISPR2/), with the parameters as default. Meanwhile, two RNA sequences (TGAACGCAGTACCGTTCGGAAGG and CTTCTGGCTTACGGGCCACGGGG) were chosen to target TIFY and CCT domain of JAZ15, respectively. The fragments were inserted into a CRISPR/Cas9 vector pCAMBIA3301, which contains a ZmU61 promoter and a maize UBI promoter to drive the expression of single guide RNA (GTTTTAGAGCTAGAAATAGCAAGTTAAAATAAGGCTAGTCCGTTATCAACTTGAAAAAGTGGCACCGAGTCGGTGC) and Cas9, respectively. The constructs were verified by resequencing and all transformants were identified by PCR. The primers for both targeting sites of *COI1a* and *JAZ15* are as follows, respectively. *COI1a*: F1/R1: AGGATGAAGGGAGTGAATGGC/CAGTTCCTCAGTAGGGCACG, F2/R2: GTGCTTACGTGGCGTGAAAC/TGAACACGACTACACGACGA; *JAZ15*: F1/R1: TATCCTCACGCACCAGGTTT/TCTGCTGGCTAAGAGCATGAG, F2/R2: GGCTAGAGTCACTCCGGTCT/GCTCTCGGAGCCTGACTTAC.

*Agrobacterium tumefaciens strain* EHA105 harboring the resulting binary plasmids was used to transform immature embryos of receptor inbred KN5585. Transformed embryos were cultured on the co-culture medium in dark for 3 days at 23 °C, then transferred to substitution medium for 6 days at 28 °C, followed by another 4-week selection in selection medium containing the herbicide Bialaphos (Bar). Herbicide-resistant calluses were selected and transferred to generation medium and cultured for 3 weeks at 25 °C with a 5000 lux light condition, followed by subsequent generation of T_0_ transgenic seedlings in a greenhouse after approximately 4 months. The positive transgenic plants were identified by sequencing the target regions of both *COI1a* and *JAZ15* chosen to be self-pollinated to generate T1 plants.

### 4.8. JA Signaling Pathway Gene Family Identification and Bioinformatics Analysis

The protein sequences of COIs, JAZs in Arabidopsis and rice were downloaded from the Arabidopsis Information Resource (http://www.Arabidopsis.org/), the rice genome annotation database (http://rice.plantbiology.msu.edu/), respectively, then used to BLAST genome data of maize (ver. 4.0) in NCBI (https://blast.ncbi.nlm.nih.gov/Blast.cgi), with E value less than 1.0 × 10^−05^. For all non-redundant sequences of JAZs, genes lacking typical TIFY or Jas gene functional domains were ruled out. Comparisons were made using the online site Gramene (http://www.gramene.org/) to determine the location of each *ZmJAZ* and *ZmCOI* gene, as well as the coding sequences and genome sequences of maize ver. 4.0. The protein sequences of MYC2, MYC3, and MYC4 in Arabidopsis were obtained from TAIR (https://www.arabidopsis.org/). Three homologous *ZmbHLH* genes were found by BLASTing their amino acid sequences in Gramene. All putative candidates were manually verified with Pfam (http://pfam.xfam.org/) to confirm the presence of conserved protein domains.

To illustrate the structures of *ZmJAZ* and *ZmCOIs* genes, the coding sequence of each gene was aligned with its genomic sequence using the Gene Structure Display Server (GSDS) program (http://gsds.gao-lab.org/). The position of each candidate gene in the corresponding chromosome was confirmed by using MaizeGDB database (https://www.maizegdb.org/).

Multiple Expectation Maximization for Motif Elicitation (MEME) (http://meme-suite.org/tools/meme) was used to identify the motifs of ZmJAZ and ZmCOIs proteins with default settings. Protein domains and significant sites were identified using NCBI (https://www.ncbi.nlm.nih.gov/) and Pfam (http://pfam.xfam.org/). DNAMAN and Weblogo (http://weblogo.berkeley.edu/logo.cgi) were used to perform multi-sequence alignment between ZmJAZs and ZmCOIs genes with that in other plant species, and ZmCOIs with F-box-like proteins in Human (*Homo sapiens*) and Zebrafish (*Danio rerio*). A phylogenetic tree was constructed according to the neighbor-joining method using the MEGA6.0 program.

## 5. Conclusions

In summary, using a combination of transcriptome, genome-wide screening and biochemical analysis, we investigated systemically the maize COIs-JAZ-MYC function modules that might be associated with GSR resistance. ZmCOI1a and ZmJAZ15 were successfully identified as key components involved in maize JA signaling pathways in response to *F. graminearum* infection. By deploying CRISPR-cas9 knockout and *Mutator* insertional mutants, we demonstrated that *coi1a* mutant and JA-deficient mutant *opr7-5opr8-2* are more resistant, whereas *jaz15* mutant is more susceptible to GSR, suggesting that ZmJAZ15 plays a positive role, whereas ZmCOIa and JA itself might function as susceptibility factors, in maize immunity to GSR.

## Data Availability

Not applicable.

## Supplementary Materials

The following are available online at https://www.mdpi.com/1422-0067/22/2/870/s1. Figure S1. RNA-seq analysis of MeJA-treated B73 stems. Figure S2. Selected genes involved in JA biosynthetic and other oxylipin pathways impacted by MeJA treatment. Figure S3. Gene structures of ZmCOIs. Figure S4. The chromosol locations of ZmCOIs and ZmJAZs genes. Figure S5. Phylogenetic relationship of ZmCOIs. Figure S6. The conserved protein motifs in ZmCOIs. Figure S7. Conserved domains in ZmCOIs. Figure S8. Mutation of A at position to V in ZmCOI1a. Figure S9. Interaction between COIs and selected JAZs in the presence of COR at 100µM. Figure S10. Targeted mutagenesis of COI1a using CRISPR-cas9. Figure S11. Effect of exogenous JA on the root growth of WT (KN5585) and CRISPR-cas9 KO mutants of ZmCOI1a. Figure S12. Targeted mutagenesis of JAZ15 using CRISPR-cas9. Table S1. The original data of RNA-seq. Table S2. DEGs upon treatment with MeJA. Table S3. Information of all maize JAZ and COI genes. Table S4. Sequence alignment of bHLH99, bHLH91 and MYC7 to MYC2, MYC3 and MYC4 in Arabidopsis. Table S5. Information of primers used in this study.

## Abbreviations

12-OPDA	12-Oxo-Phytodienoic Acid
bHLH	basic Helix-Loop-Helix
COI	Coronatine-Insensitive
COR	Coronatine
DEGs	Differently Expressed Genes
GSR	Gibberella Stalk Rot
PD	Pull-Down
JA	Jasmonic Acid
JAs	Jasmonates
JA-Ile	JA-Isoleucine
JAZ	Jasmonate-Zim domain
LOXs	Lipoxygenases
MeJA	Methyl Jasmonic Acid
OPRs	12-Oxo-Phytodienoic Acid Reductases
TF	transcription facor
Y2H	Yeast-Two-Hybrid
