# Supplementary material for: Genome-Wide Characterization of Jasmonates Signaling Components Reveals the Essential Role of ZmCOI1a-ZmJAZ15 Action Module in Regulating Maize Immunity to Gibberella Stalk Rot
Source: Int J Mol Sci. 2021 Jan 16;22(2):870. doi: 10.3390/ijms22020870 (PMC7830991; doi:10.3390/ijms22020870)
Supplement: Supplementary file 1 [file ijms-22-00870-s001.zip › Supplementary files-20201222/Legends for Supplementary materials-20201222.docx]

**Legends for Supplementary materials**

**Supplemental Figure 1. RNA-seq analysis of MeJA-treated B73 stems.** A, The correlation between sample replicates for differentially expressed genes (DEGs) with a selection criteria at *padj*<0.05 and log2Foldchange>1; B, The total number of up- and down-regulated DEGs at 6 and 24 hpt after spray with MeJA. Red and blue represents the number of up-regulated and down-regulated genes, respectively; C, Venn diagram displaying number of DEGs between samples at 6 hpt and 24 hpt. D, Volcano diagram shows the distribution of DEGs for 6 hpt (left) and 24 hpt (right), respectively. Blue dots and red dots represent the down-regulated and up-regulated genes, whereas gray ones for genes without changes, respectively. The Log10 on Y-axis indicates the p-value and X-axis in log2 does for fold changes in MeJA treated samples relative to control.

**Supplemental Figure 2. Selected genes involved in JA biosynthetic and other oxylipin pathways impacted by MeJA treatment.** A, lipoxygenases pathway; B-D, JA biosynthetic pathway; E, SA signaling pathway. The upregulated genes were highlighted in red and downregulated were in blue, whereas the light colors indicate less changed.

**Supplementary Figure 3. Gene structures of ZmCOIs (A) and ZmJAZs (B)**. Black squares represent the exons, while introns are indicated by dotted lines, and solid lines stand for UTRs.

**Supplementary Figure 4. The chromosol locations of ZmCOIs and ZmJAZs genes.** The relative genomic length is indicated at the bottom of chromosomes.

**Supplementary Figure 5. Phylogenetic relationship of ZmCOIs (A) and ZmJAZs (B) proteins with other species**. A, ZmCOI1s with the COIs from selected plant species and F-box-like proteins from Human (*Homo sapiens*) and Zebrafish (*Danio rerio*). B, ZmJAZs with the JAZs selected plant species. At, *Arabidopsis thaliana*; Hs: Homo sapiens; Mp, *Marchantia polymorpha*; Os, *Oryza sativa* ; Sb, *Sorghum bicolor*; Si, *Setaria italica*; Zm*, Zea mays*.

**Supplementary Figure 6. The conserved protein motifs in ZmCOIs (A) and ZmJAZs (B) proteins**. The colored boxes indicate different motifs.

**Supplementary Figure 7. Conserved domains in ZmCOIs (A) and ZmJAZs (B)**. The conserved residues were highlighted in different colors. The red square indicate the difference of ZmJAZ19 domains compared to others.

**Supplementary Figure 8. Mutation of A at position to V in ZmCOI1a.** The mutated residue in ZmCOI1^A388V^ compared to WT ZmCOI1a was highlighted by red box.

**Supplementary Figure 9. Interaction between COIs and selected JAZs in the presence of COR at 100µM.**

**Supplementary Figure 10. Targeted mutagenesis of COI1a using CRISPR-cas9.** A, Structure of ZmCOI1a gene on chromosomes 3 and two gRNAs target sites designed to generate DSBs in F-BOX motif (blue bar inside first exon) and on the LRR domain. The sequences of double strands of the gene are shown in black box, in which the gree indicates the sense strand. The first nt (in bold) in the bigining of 20 bp of gRNAs (above the double-strand box for ZmCOI1a) was changed to G., red nucleotides indicate PAM sequences for the gRNAs. B-D, the sequences from selected T0 plants with site-specific mutations accompanied by corresponding regions of the sequencing chromatograms. The description above the sequences is the mutation allele obtained, represented by the number in parentheses. The nucleotide changes are shown in the sequences (highlighted in blue). The mutation of deletion or insertion are also indicated using “-” or “+” in front of the number on the right side of each sequence.

**Supplementary Figure 11. Effect of exogenous JA on the root growth of WT (KN5585) and CRISPR-cas9 KO mutants of ZmCOI1a.** A, WT and mutants seeds were surface sterilized and transferred to the agar medium containing 20 mM of JA and kept in a condition of 16h light/8h dark at 28 ^o^C. The pictures were taken and the roots were measured at 6d post germination. B, quantification of the length of WT and KO mutant roots. The percentage on the side indicates the reduction rate of JA-treated roots over control in each line. The statistical analysis was analyzed by two-way ANOVA. The different letters above the lines indicate significant differences for the root reduction ratio between different genotypes, and that above the bars indicate significant differences between Ctl. and JA treatment within same genotype, at p<0.01 level, respectively. The same letters on the bars indicate “not significant” within same treatment between different lines. Ctl., control without JA treatment.

**Supplementary Figure 12. Targeted mutagenesis of JAZ15 using CRISPR-cas9.** A, Structure of JAZ15 gene on chromosomes 7 and two gRNAs target sites designed to generate DSBs in F-BOX motif (blue bar inside first exon) and on the CCT domain. The sequences of double strands of the gene are shown in black box, in which the gree indicates the sense strand. The first nt (in bold) in the bigining of 20 bp of gRNAs (above the double-strand box for ZmJAZ15) was changed to G., red nucleotides indicate PAM sequences for the gRNAs. B and C, the sequences from selected T0 plants with site-specific mutations accompanied by corresponding regions of the sequencing chromatograms. The description above the sequences is the mutation allele obtained, represented by the number in parentheses. The nucleotide changes are shown in the sequences (highlighted in blue). The mutation of deletion or insertion are also indicated using “-” or “+” in front of the number on the right side of each sequence.

**Supplementary Table S1. The original data of RNA-seq.**

**Supplementary Table S2. DEGs upon treatment with MeJA.**

**Supplementary Table S3. Information of all maize JAZ and COI genes.**

**Supplementary Table S4. Sequence alignment of bHLH99, bHLH91 and MYC7 to MYC2, MYC3 and MYC4 in Arabidopsis.**

**Supplementary Table S5. Information of primers used in this study**
