# Supplementary figures and images for: Genome-Wide Characterization of Jasmonates Signaling Components Reveals the Essential Role of ZmCOI1a-ZmJAZ15 Action Module in Regulating Maize Immunity to Gibberella Stalk Rot
Source: Int J Mol Sci. 2021 Jan 16;22(2):870. doi: 10.3390/ijms22020870 (PMC7830991; doi:10.3390/ijms22020870)

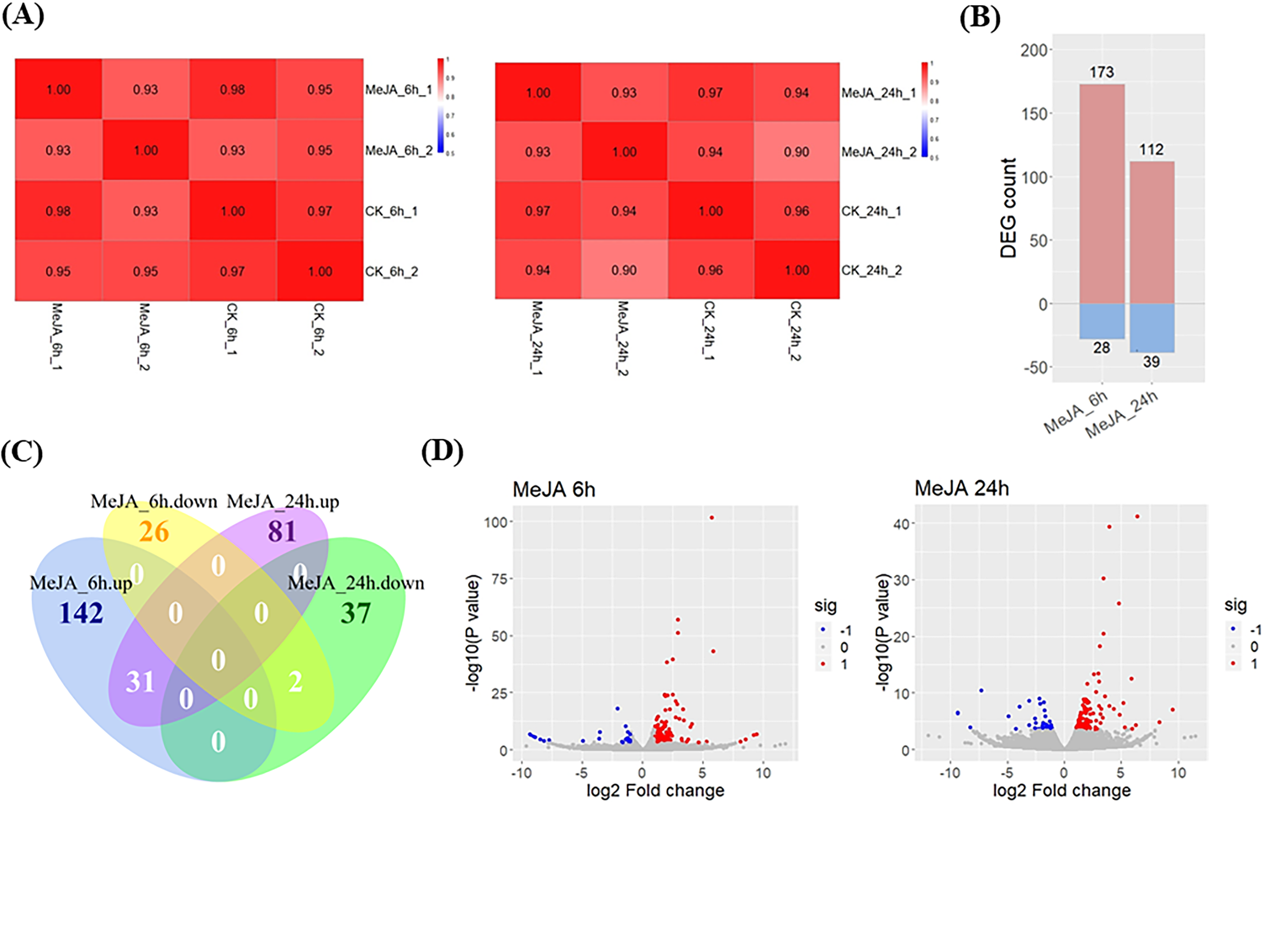

Supplement: Supplementary file 1 [file ijms-22-00870-s001.zip › Supplementary files-20201222/Figure S1.tif]

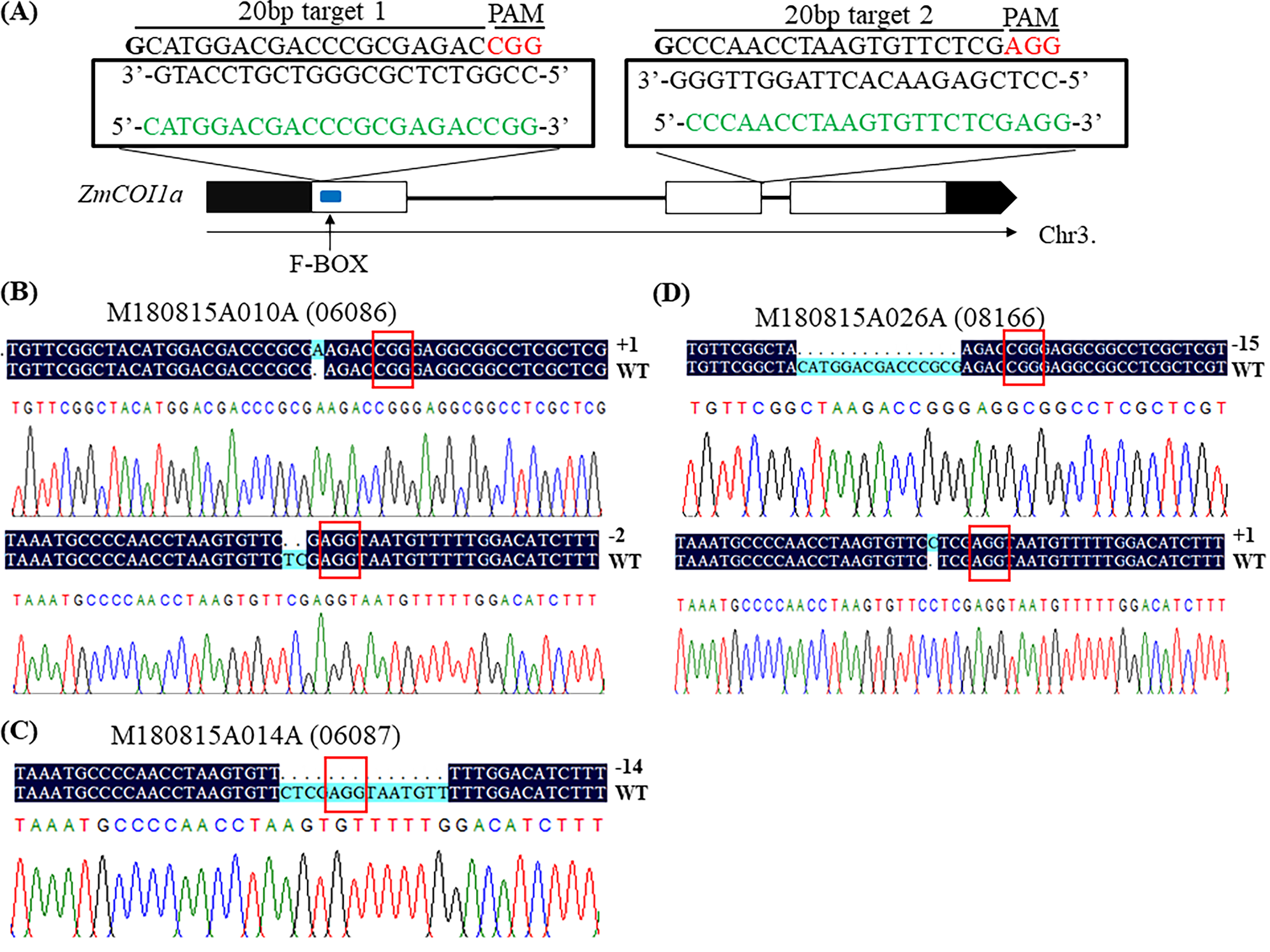

Supplement: Supplementary file 1 [file ijms-22-00870-s001.zip › Supplementary files-20201222/Figure S10.tif]

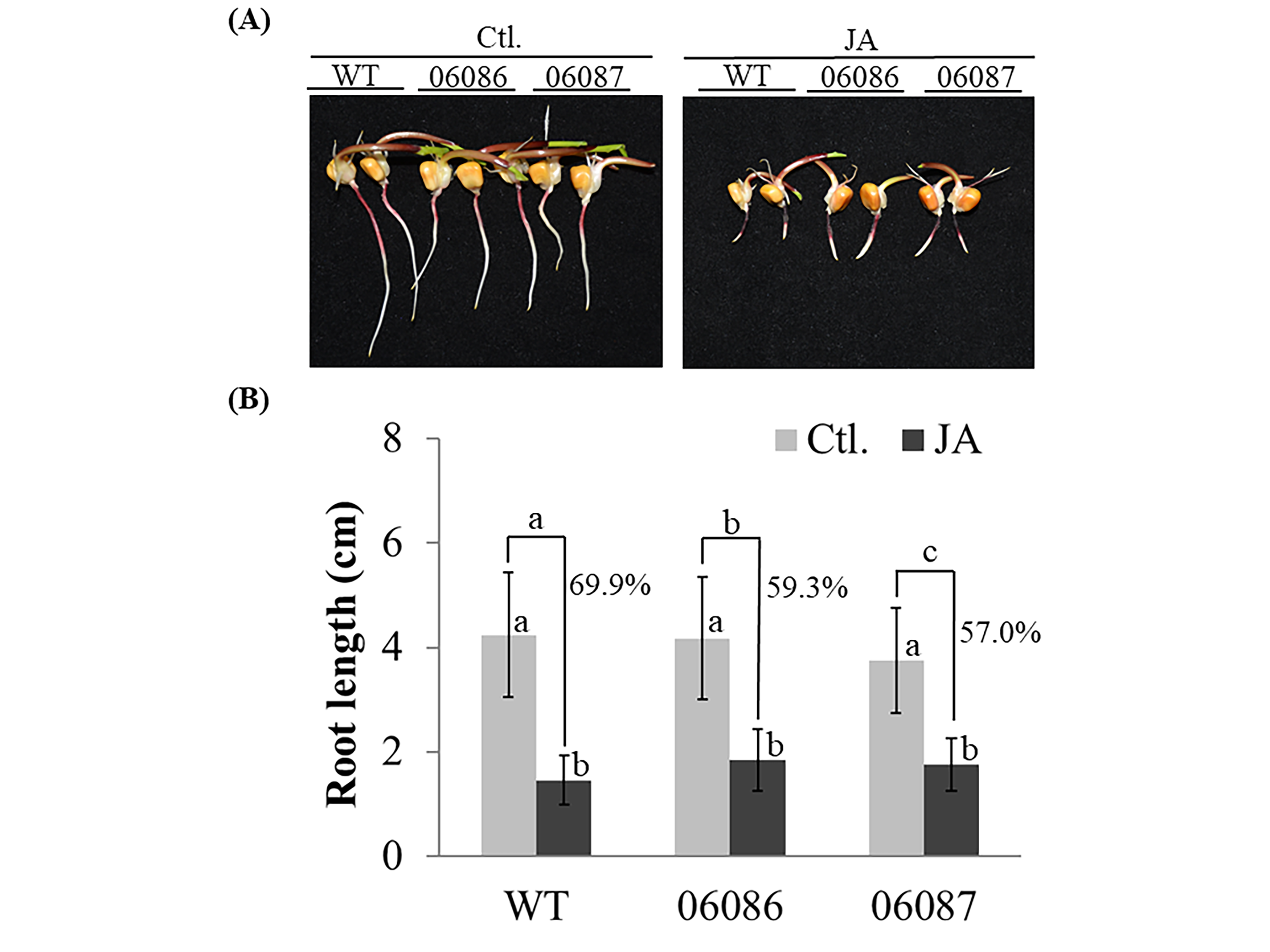

Supplement: Supplementary file 1 [file ijms-22-00870-s001.zip › Supplementary files-20201222/Figure S11.tif]

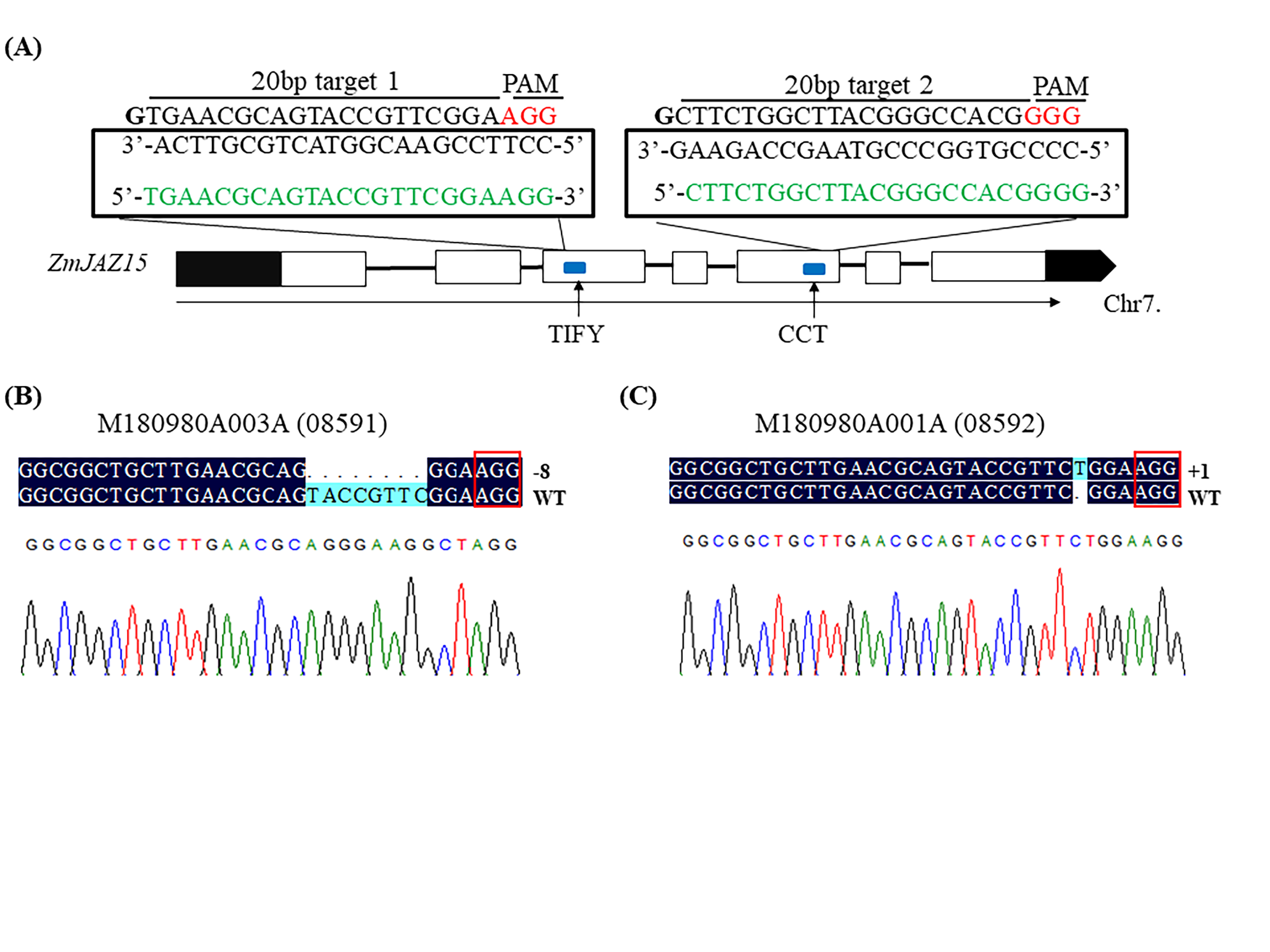

Supplement: Supplementary file 1 [file ijms-22-00870-s001.zip › Supplementary files-20201222/Figure S12.tif]

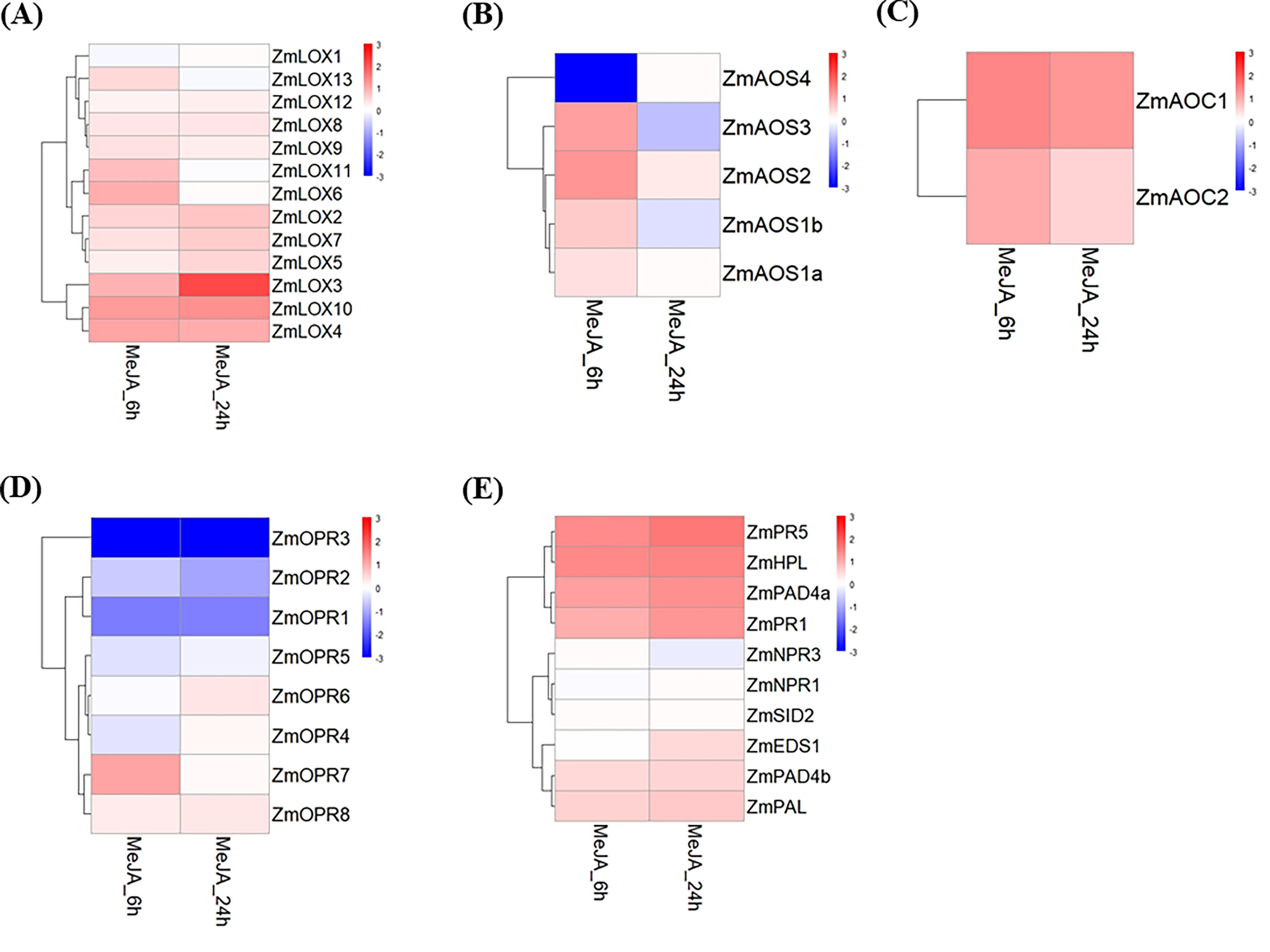

Supplement: Supplementary file 1 [file ijms-22-00870-s001.zip › Supplementary files-20201222/Figure S2.tif]

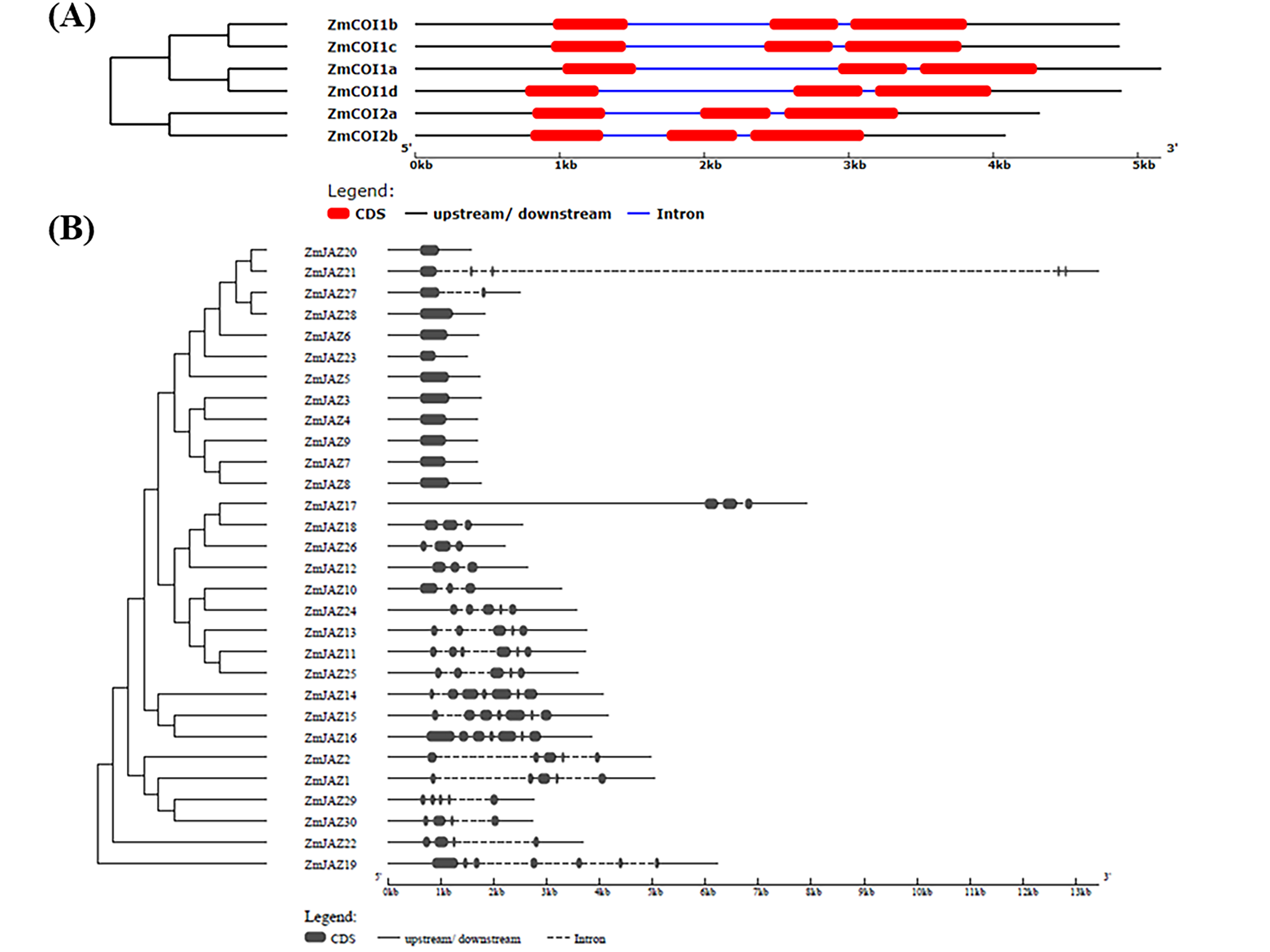

Supplement: Supplementary file 1 [file ijms-22-00870-s001.zip › Supplementary files-20201222/Figure S3.tif]

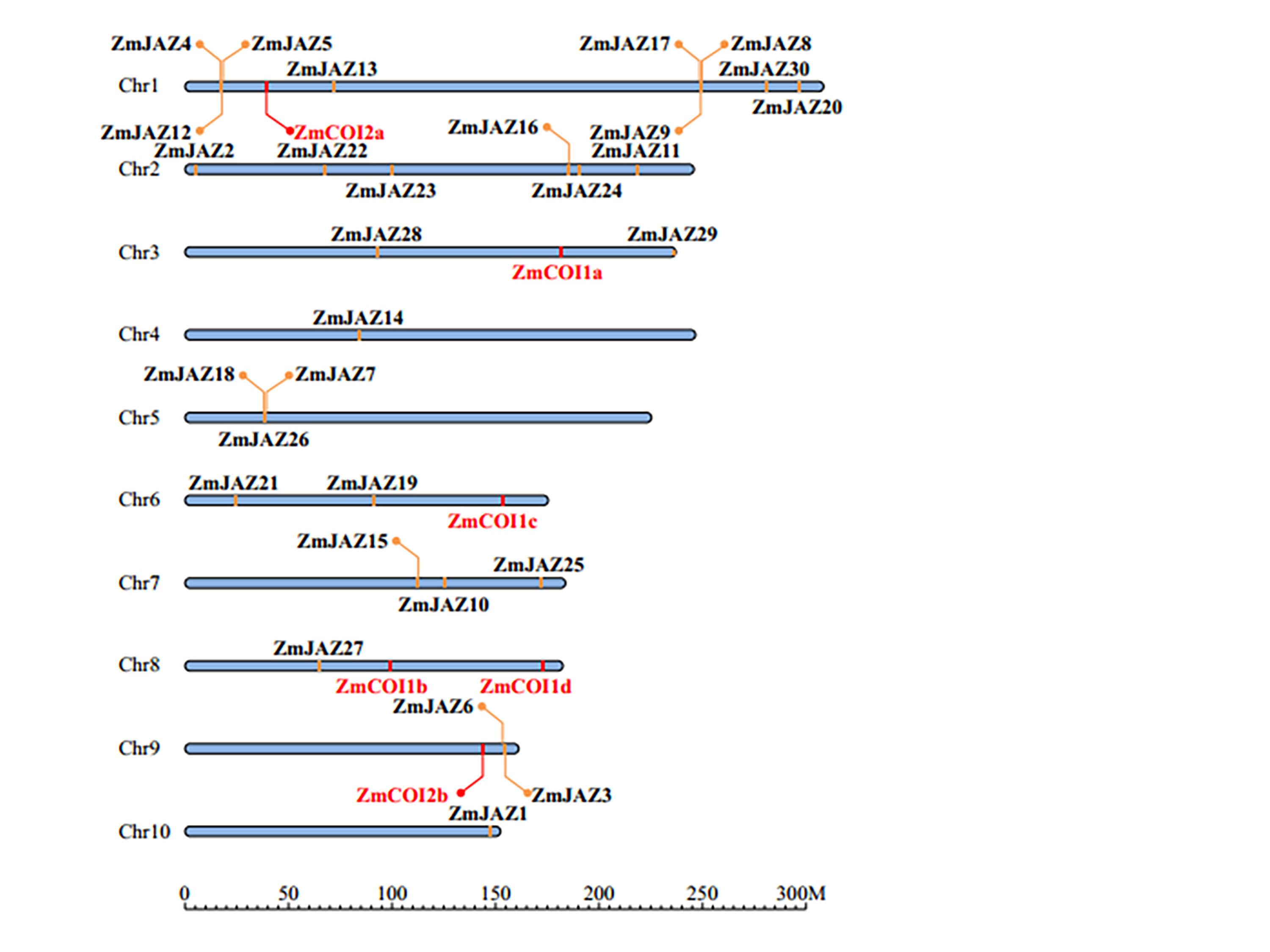

Supplement: Supplementary file 1 [file ijms-22-00870-s001.zip › Supplementary files-20201222/Figure S4.tif]

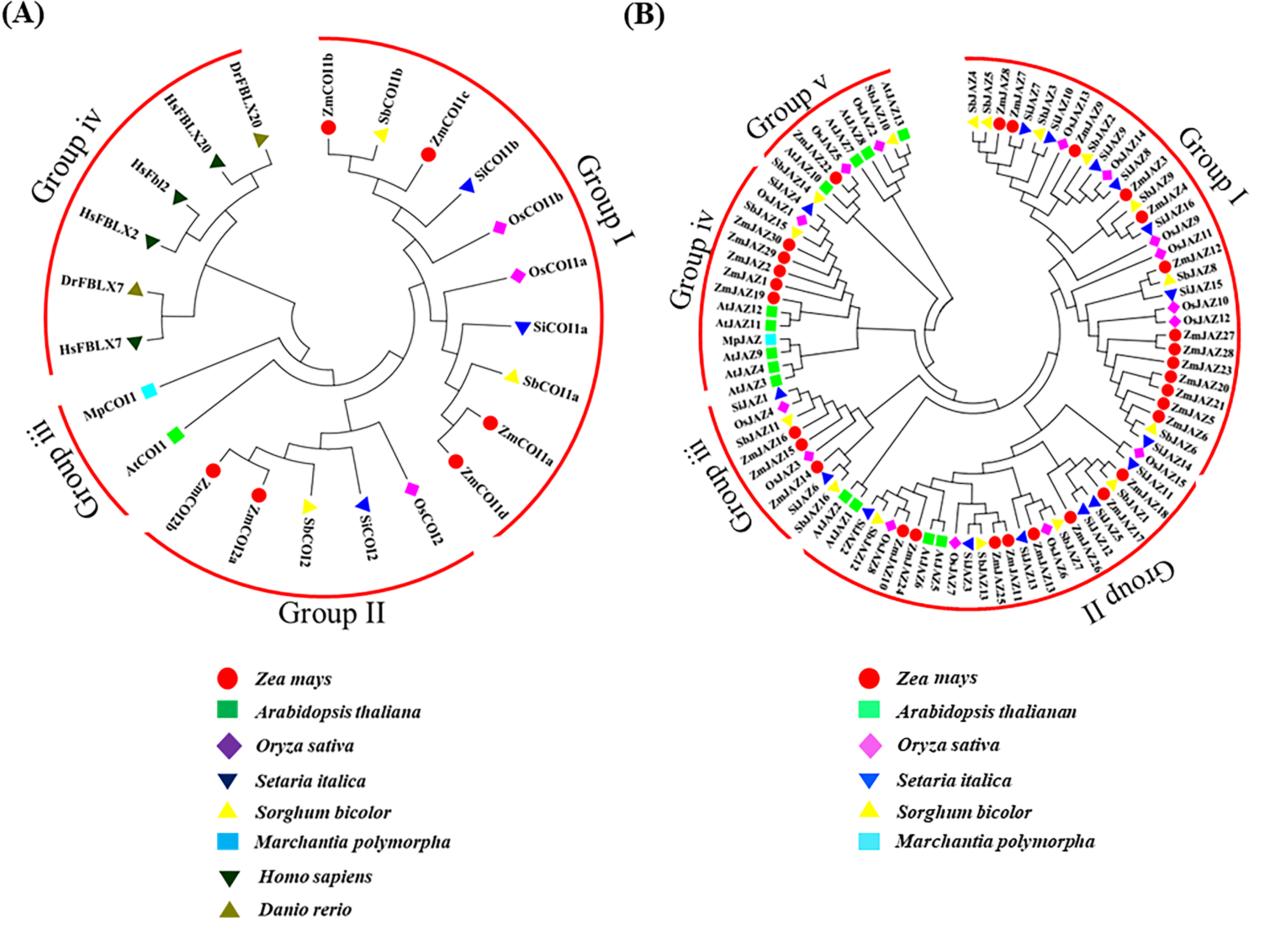

Supplement: Supplementary file 1 [file ijms-22-00870-s001.zip › Supplementary files-20201222/Figure S5.tif]

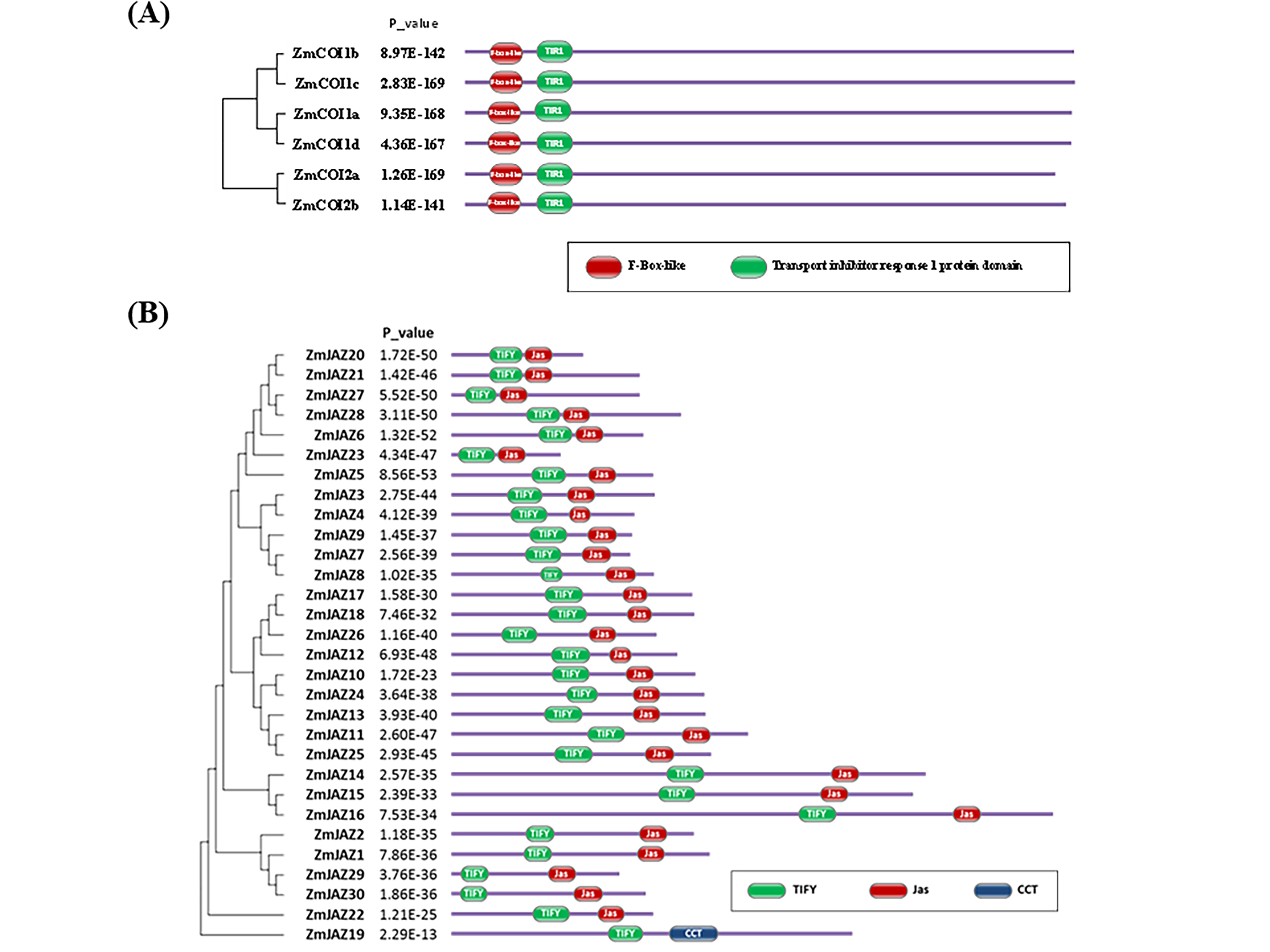

Supplement: Supplementary file 1 [file ijms-22-00870-s001.zip › Supplementary files-20201222/Figure S6.tif]

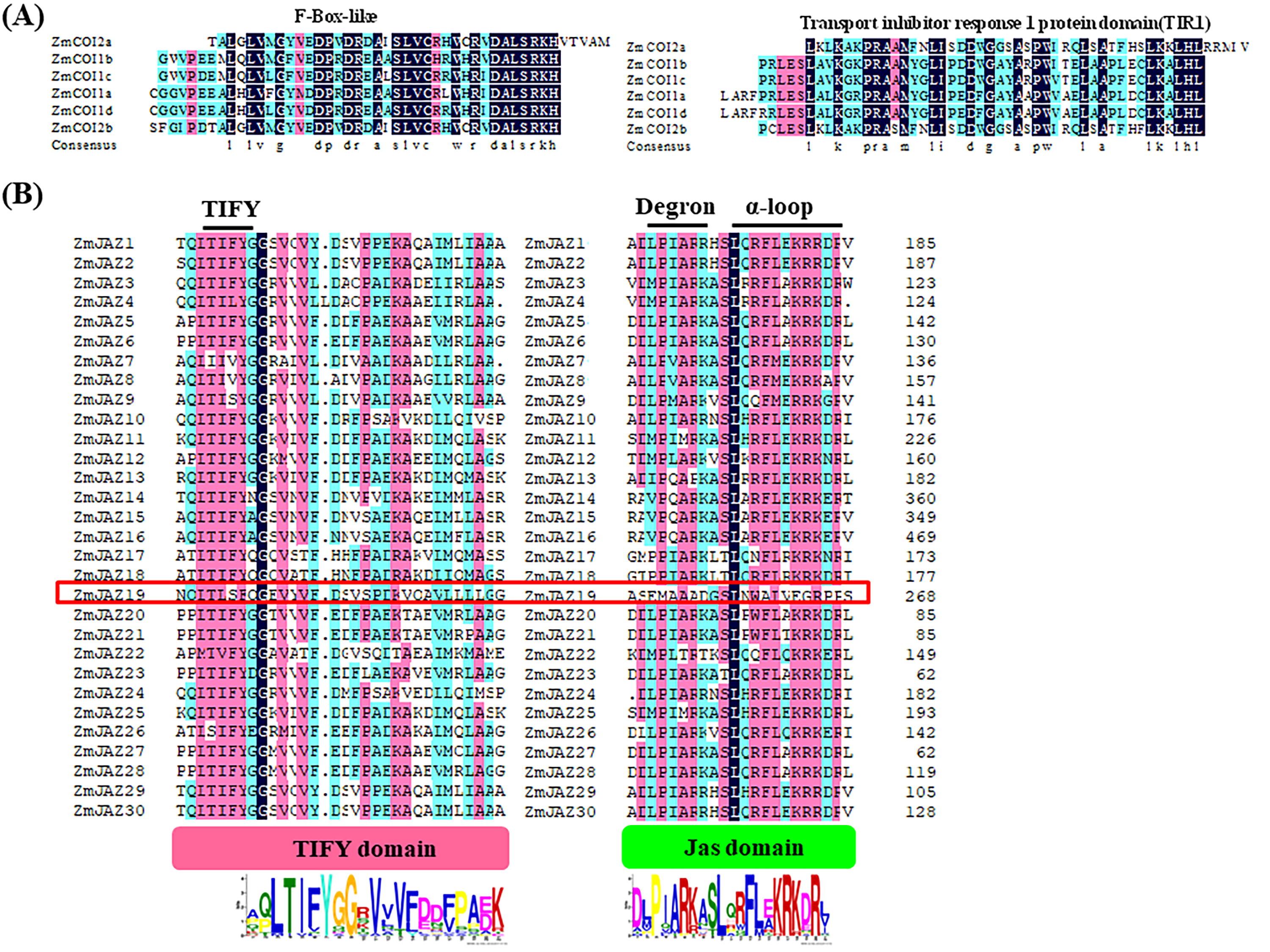

Supplement: Supplementary file 1 [file ijms-22-00870-s001.zip › Supplementary files-20201222/Figure S7.tif]

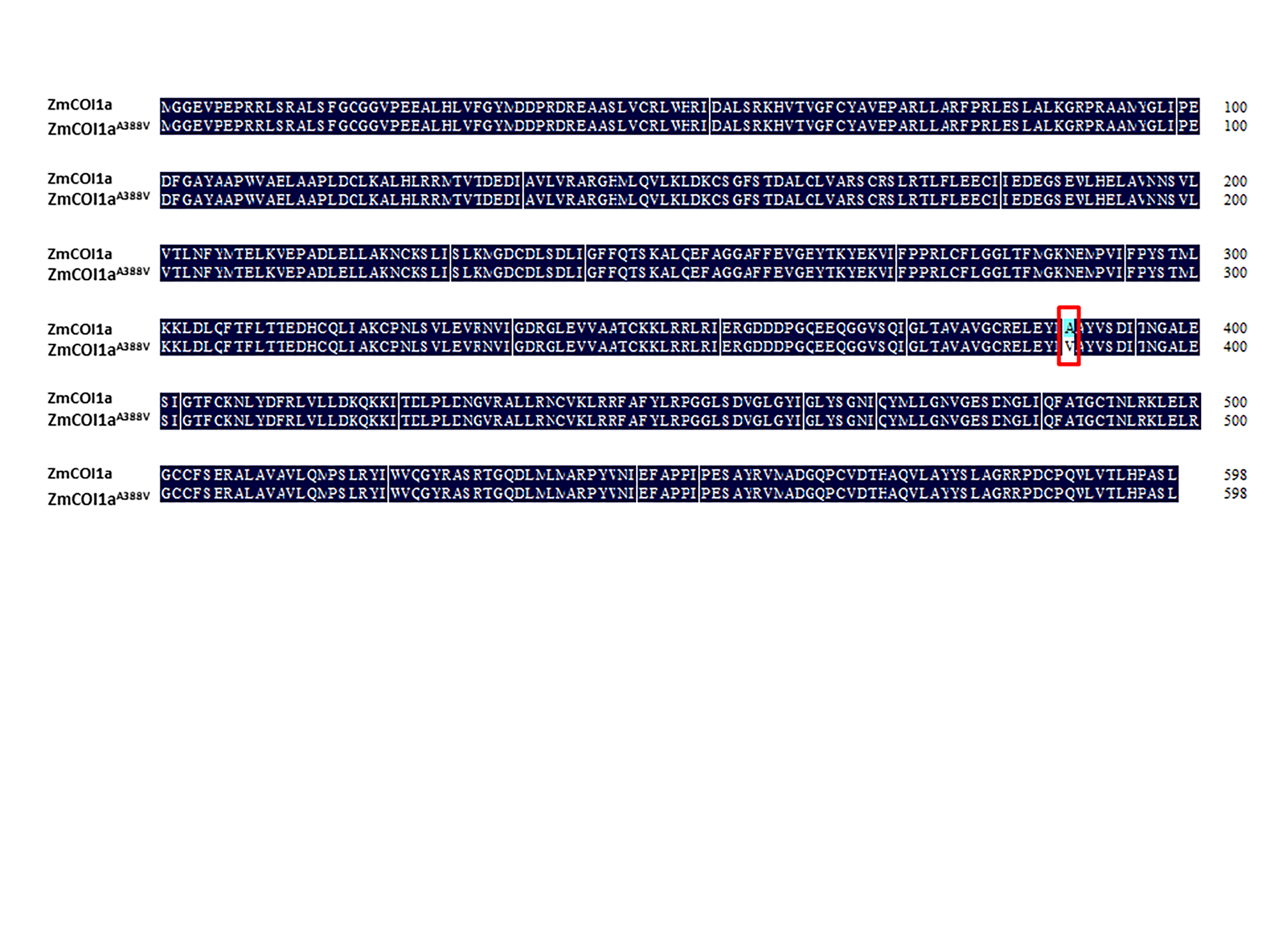

Supplement: Supplementary file 1 [file ijms-22-00870-s001.zip › Supplementary files-20201222/Figure S8.tif]

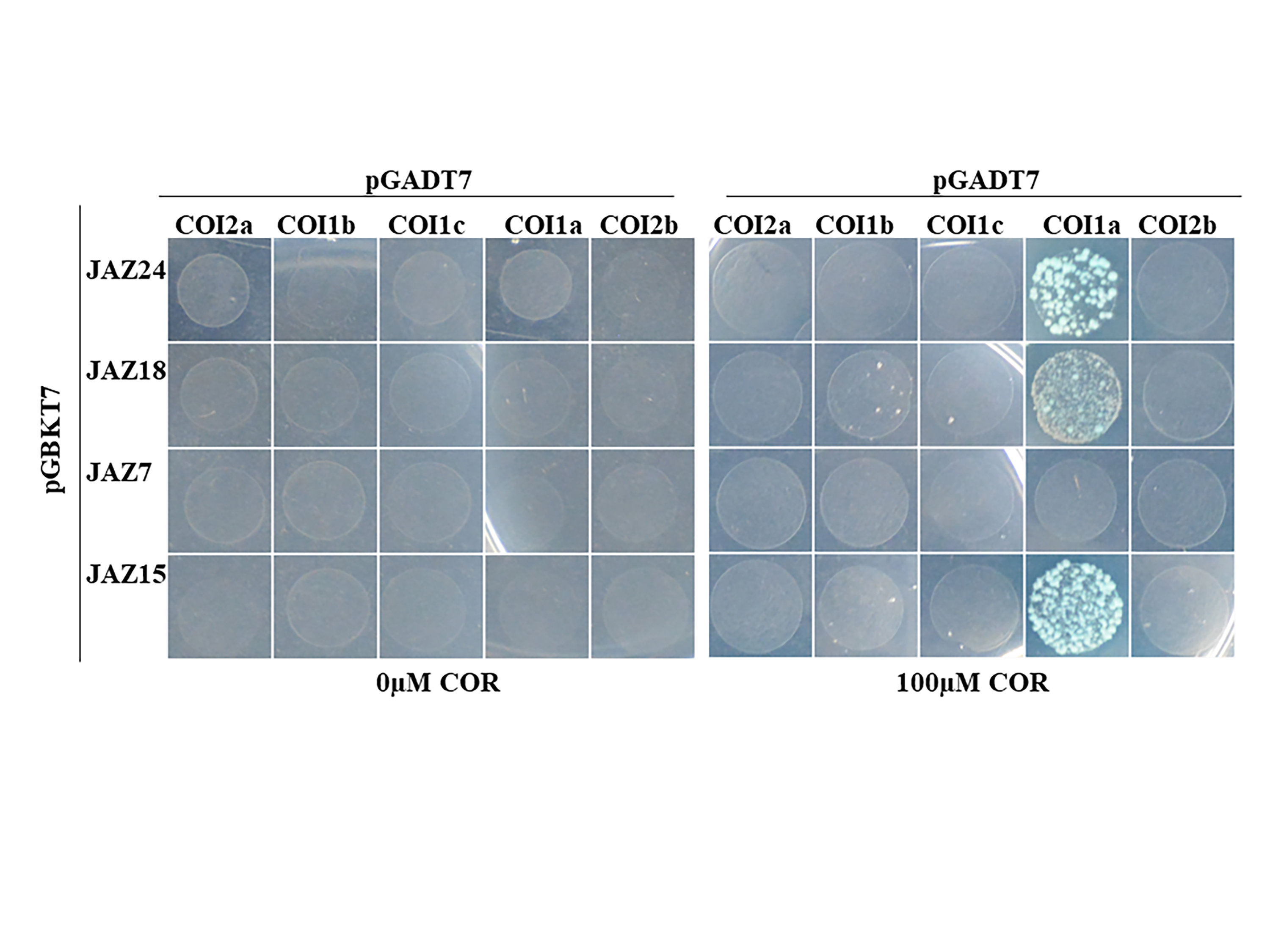

Supplement: Supplementary file 1 [file ijms-22-00870-s001.zip › Supplementary files-20201222/Figure S9.tif]
